# Molecular phylogeny of heritable symbionts and microbiota diversity analysis in phlebotominae sand flies and *Culex nigripalpus* from Colombia

**DOI:** 10.1371/journal.pntd.0009942

**Published:** 2021-12-20

**Authors:** Rafael J. Vivero-Gomez, Víctor A. Castañeda-Monsalve, María Claudia Atencia, Richard Hoyos-Lopez, Gregory D. Hurst, Gloria Cadavid-Restrepo, Claudia Ximena Moreno-Herrera

**Affiliations:** 1 Grupo de Microbiodiversidad y Bioprospección, Laboratorio de Biología Celular y Molecular, Universidad Nacional de Colombia sede Medellín, Medellín, Colombia; 2 Doctorado Microbiología Tropical, Universidad de Córdoba, Montería, Colombia; 3 Departamento de Biología, Universidad de Córdoba, Montería, Colombia; 4 Institute of Infection, Veterinary and Ecological Sciences, University of Liverpool, Liverpool, United Kingdom; All India Institute of Medical Science - Bhopal, INDIA

## Abstract

**Background:**

Secondary symbionts of insects include a range of bacteria and fungi that perform various functional roles on their hosts, such as fitness, tolerance to heat stress, susceptibility to insecticides and effects on reproduction. These endosymbionts could have the potential to shape microbial communites and high potential to develop strategies for mosquito-borne disease control.

**Methodology/Principal findings:**

The relative frequency and molecular phylogeny of *Wolbachia*, *Microsporidia* and *Cardinium* were determined of phlebotomine sand flies and mosquitoes in two regions from Colombia. Illumina Miseq using the 16S rRNA gene as a biomarker was conducted to examine the microbiota. Different percentages of natural infection by *Wolbachia*, *Cardinium*, and *Microsporidia* in phlebotomines and mosquitoes were detected. Phylogenetic analysis of *Wolbachia* shows putative new strains of *Lutzomyia gomezi (wLgom)*, *Brumptomyia hamata* (*wBrham*), and a putative new group associated with *Culex nigripalpus (Cnig)* from the Andean region, located in Supergroup A and Supergroup B, respectively. The sequences of *Microsporidia* were obtained of *Pi*. *pia* and *Cx*. *nigripalpus*, which are located on phylogeny in the IV clade (terrestrial origin). The *Cardinium* of *Tr*. *triramula* and *Ps*. *shannoni* were located in group C next to *Culicoides* sequences while *Cardinium* of *Mi*. *cayennensis* formed two putative new subgroups of *Cardinium* in group A. In total were obtained 550 bacterial amplicon sequence variants (ASVs) and 189 taxa to the genus level. The microbiota profiles of Sand flies and mosquitoes showed mainly at the phylum level to Proteobacteria (67.6%), Firmicutes (17.9%) and Actinobacteria (7.4%). High percentages of relative abundance for *Wolbachia* (30%-83%) in *Lu*. *gomezi*, *Ev*. *dubitans*, *Mi*. *micropyga*, *Br*. *hamata*, and *Cx*. *nigripalpus* were found. ASVs assigned as *Microsporidia* were found in greater abundance in *Pi*. *pia* (23%) and *Cx*. *nigripalpus* (11%). An important finding is the detection of *Rickettsia* in *Pi*. *pia* (58,8%) and *Bartonella* sp. in *Cx*. *nigripalpus*.

**Conclusions/Significance:**

We found that *Wolbachia* infection significantly decreased the alpha diversity and negatively impacts the number of taxa on sand flies and *Culex nigripalpus*. The Principal Coordinate Analysis (PCoA) is consistent, which showed statistically significant differences (PERMANOVA, F = 2.4744; R2 = 0.18363; p-value = 0.007) between the microbiota of sand flies and mosquitoes depending on its origin, host and possibly for the abundance of some endosymbionts (*Wolbachia*, *Rickettsia*).

## Author summary

The secondary endosymbionts can positively influence the metabolism of many compounds essential for the survival of the insect vectors, provide resistance to pathogens and impact susceptibility to insecticides, as also the tolerance to heat stress. We provide information from new records of natural infection of secondary endosymbionts, such as *Wolbachia*, *Cardinium*, *Microsporidia*, *Flavobacterium*, and *Rickettsia* in phlebotomine sand flies and mosquitoes from Colombia. An important finding is the detection of Bartonella sp. in *Cx*. *nigripalpus*. Clear differences were found in the composition and diversity of microbiota at the intra-specific and interspecific levels in sand flies and *Cx*. *nigripalpus*, which may depend in the of the load of natural infection of endosymbionts (as *Wolbachia*), the geographical distribution and host.

## Introduction

A comprehensive understanding of the biology of insects requires that they must be studied in an ecological context where microorganisms are considered an important component [1], due to their essential role in disease transmission, and potential to develop strategies for mosquito-borne disease control [2]. Facultative secondary symbionts (S-symbionts) can be transmitted horizontally, vertically and perform various functional roles on their hosts, such as modulation of fitness, increasing tolerance to heat stress and susceptibility to insecticides, manipulate the reproductive properties of their insect hosts [3]. Some endosymbionts can be found in insect hemolymph and thus they can interact directly with secreted molecules of the humoral immune response. In this regard, several studies have also examined the interaction between endosymbionts infection in mosquitoes and their vector competence for important mammalian viral pathogens [4]. The insect-symbiont-pathogen interactions will lead to more efficient management strategies, particularly those involving integrated and biological control tactics, those seeking to reduce reliance on broad-spectrum insecticides [5].

S-symbionts include a range of bacteria and fungi that could include *Wolbachia*, *Cardinium*, *Spiroplasma*, *Arsenophonus*, *Rickettsia*, *Microsporidia*, and others [5]. *Wolbachia* is a common bacterial endosymbiont present in arthropods and nematodes [6].

In arthropods, *Wolbachia* typically acts as a reproductive parasite associated with various manipulations on its host [6]. Some associations between strain and hosts, show that *Wolbachia* is disseminated throughout most tissues (midgut, salivary glands, muscles, fatty body, head, malp. Tub), while other strains are much more restricted, being predominantly limited to reproductive tissues (Testes, Ovaries) [7, 8]. Its vertical and horizontal transmission has also been demonstrated [9]. *Wolbachia* was first detected in the mosquito *Culex pipiens*, and it was recently revealed that *Wolbachia* infection makes *Drosophila* fruit flies more resistant to various RNA viruses, and since then has become established as a successful biocontrol agent for many pests and disease vectors as Dengue, West Nile, Yellow fever, Zika, and Chikungunya [4, 10].

Different *Wolbachia* strains can also reduce *Plasmodium* development in *Anopheles* species [4]. Recently, it was also introduced in cell lines (LL5) and eggs of *Lutzomyia longipalpis*, to design control strategies against leishmaniasis [11–14]. In natural populations of Phlebotomine sandfly species, infections for *Wolbachia* are limited to species of *Lutzomyia*, *Micropygomyia*, *Evandromyia*, *Sciopemyia*, *Psychodopygus*, *Pintomyia*, *Psathyromyia sergentomyia*, and *Phlebotomus*, with strains belonging to A and B Supergroup [15–17]. In mosquitoes, it has a major presence in different populations of *Culex*, *Aedes*, *Mansonia*, and *Anopheles* [18, 19]. Although in Colombia there is a wide richness of insect vectors of leishmaniasis, only *Wolbachia* has been documented in *Pintomyia evansi*, *Evandromyia dubitans*, and *Micropygomyia cayennenis* [20]. A greater information gap exists in Colombia about the frequency of infection for *Wolbachia* in mosquitoes and sand flies [20, 21].

*Cardinium* members are capable also of inducing reproductive abnormalities in their hosts (mainly in wasps, mites, and spiders) [22], a low frequency of infection in arthropods (*Pintomyia evansi*, *Culex pipiens*, *Aedes albopictus*, *Culicoides*) with medical importance is brought in the literature [23–26]. Since its discovery, *Cardinium* has been found in four orders and approximately 6%–7% of arthropods and now causes three of the four classic phenotypes often associated with reproductive parasites: cytoplasmic incompatibility, feminization and parthenogenesis [27, 28]. Several studies have reported that multiple infections of *Wolbachia-Cardinium* occur commonly in several arthropod species (*Drosophila simulans*, *Aedes albopictus*, *Nasonia vitripennis*, *Callosobruchus chinensis*, *Tetranychus cinnabarinus*) [29–32], indicating that recently, we found multiple infection patterns in natural populations of *Pintomyia evansi* from Colombia in the intestinal microbiome [23], being the only report for sand flies. In contrast with the two more widespread reproductive parasites, *Wolbachia* and *Spiroplasma*, are far less known of causing infections with *Cardinium* and possible consequences in the Diptera [33]. There are no systematic studies in Colombia on the specific detection of *Cardinium*, including the phylogenetic location of its strains and their potential distribution in sand flies and mosquitoes. This demonstrates the need for studies to suggest the potential role of *Cardinium* in vector insects.

*Spiroplasma* genus includes species that are harmless commensals, mutualists, reproductive parasites, and pathogens associated with various arthropod hosts and plants [34, 35]. Recent studies have shown that *Spiroplasma* is present in more than 4%–7% of all insect species [36, 37]. Some studies recently showed that *Spiroplasma* can protect its insect host from infections with pathogenic organisms and therefore is a potentially useful tool for the control of vector-borne diseases [38, 39]. *Spiroplasma* has been isolated in several species of *Anopheles* [40], *Aedes* [41], *Culex* [42], and recently in *Phlebotomus* sand flies [43], but *Spiroplasmas* also have a high presence in other insect orders as Hemiptera, Homoptera, Hymenoptera, Lepidoptera and Odonata [35–44]. The advancement of a symbiont-based transmission-blocking strategy for malaria, arboviral diseases and leishmaniasis requires the identification and study of symbionts as *Spiroplasma*.

The role of *Rickettsia* in heat tolerance and increased susceptibility to insecticides are well documented [45]. It was found that, in the presence of *Rickettsia*, the whitefly’s susceptibility to five out of the six insecticides tested was increased, in spite of their variable mode of action and target stages [46]. One possibility is that *Rickettsia*, like other intestinal symbionts, may be involved in the detoxification of natural and synthetic poisons. It was also reported to play an important role in increasing fecundity, survival to adulthood, and reduction in development time [47]. *Rickettsia* in different studies has been specially registered for *Phlebotomus chinensis* [48] and mosquitoes (*Anopheles*, *Culex*, and *Aedes*), but mainly in China [49], Africa [50], and the USA [51].

Microorganisms that inhabit arthropod reproductive tissues represent an exceedingly broad group, spanning many orders of bacteria, protists, viruses, and fungi as *Microsporidia* [44]. The species’ diversity of *Microsporidia*, seasonal occurrence, and transmission mechanisms remain poorly understood [52–56]. *Microsporidia* are single-celled eukaryotic microorganisms that have small genomes in the size range of prokaryotic cells and are now thought to be highly evolved fungi [57]. Molecular and morphological characterization of *Microsporidia* in sand flies, it is limited to laboratory populations and during routine dissections of *Leishmania* promastigotes in species of *Lutzomyia* and *Phlebotomus* [58, 59], but constitute the most common to black fly pathogens [57] and host as *Culex*, *Aedes*, *Anopheles*, *Ochlerotatus* mainly of *Amblyospora*, *Hazardia*, *Nosema and Culicospora* integrating the main clade from Parasites of Culicidae [58]. Recently, a microsporidian (*Microsporidia MB*) blocks *Plasmodium falciparum* transmission in *Anopheles arabiensis* mosquitoes [59]. *Microsporidia* in mosquitoes and sand flies are excellent model systems for conducting both basic and applied studies.

Many studies have focused on the influence of host–endosymbiont interactions on transmission and immunity, but interest is also emerging in the impact of endosymbiont–microbiome interactions in insects’ vector on the transmission capacity of pathogens as arbovirus, parasites and pathogenic bacteria [60]. Such founding microorganisms could have the potential to shape downstream microbial community assembly and composition via positive or negative interactions with other microorganisms and therefore may be important determinants for the offspring and transmission of tropical diseases [61, 62].

The prevalence of S-symbionts varies depending upon the host population, geographical location and host plants. This study determined the relative frequency of infection and molecular phylogeny of *Wolbachia*, *Cardinium*, *Microsporidia*, *and Spiroplasma* in natural populations of Phlebotominae sand flies and some species of mosquitoes (*Anopheles* and *Culex*) in several locations from Colombia. It also evaluated the dynamics of microbiota using MiSeq Illumina using the total DNA of natural populations.

## Material and methods

### Ethics statement

The sand flies collection was performed following the guidelines of the Colombian decree N° 1376. No specific permits were required for this study. This study was conducted under the Ministry of Environment and Sustainable Development (MADS) permission contract No. 121 of 2016, OTROSí No-25, for access to genetic resources and its derivative products. Also, the insects were collected under resolution No. 0207 of 090320 of the Ministry of Environment and Sustainable Development (MADS) and Universidad Nacional de Colombia. Sand flies were collected on private property and permission was received from landowners before sampling.

### Origin, survey, and identification of sand flies and mosquitoes

**Fig 1:**
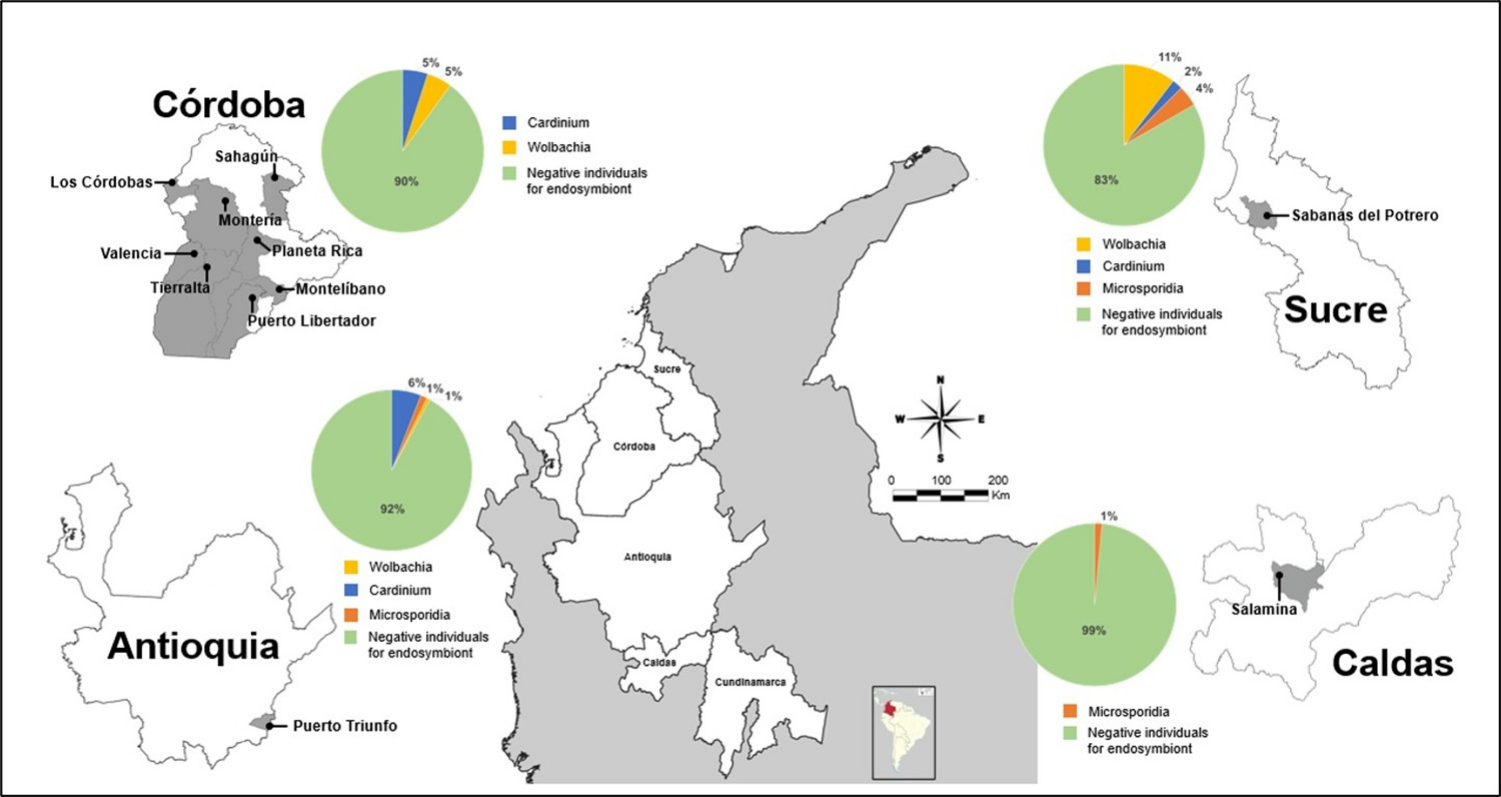
Geographic locations of sampling sites and proportion by department of insect vectors (Phlebotomine sand flies and mosquitoes) infected with *Wolbachia*, *Cardinium* and Microsporidia in Colombia. Link to the base layer of the map used: https://www.colombiaenmapas.gov.co/?e=-74.7117818345732,0.23307652371168444,-62.73668417832639,8.735414279337139,4686&b=dark-gray&u=0&t=2901&servicio=610.

Each locality was selected based on the incidence of leishmaniasis, malaria, and several arboviruses in Colombia during the last four years and historical records of adult phlebotomines and mosquitoes (*Culex* and *Anopheles*) [63–66]. Sand flies and mosquitoes were collected in peri-urban locations and natural reserves of the Andean (Antioquia, Caldas, and Cundinamarca departments) and Caribbean regions (Sucre and Cordoba departments) during an entomological survey performed between the high rainfall periods from September to November 2018 and the months from March to May 2019, with a collection frequency of three days per study area (Fig 1) using CDC light traps, located indoors and near houses, overnight, between 17:00 and 06:00 h. Shannon traps were also used for collection near houses. Additionally, a diurnal collection using a mouth aspirator was done in the surroundings of nocturnal trapping sites [67].

Collected specimens were kept dry in 1.5 mL vials and transported to the laboratory with dry ice. Once at the laboratory, they were kept at −20°C. The head and last three abdominal segments of sand flies were removed from the specimens to perform the recognition of sex and taxonomic identification following the classification system of Galati, 2018 [68]. The thorax and remaining abdominal segments were stored at −20°C until DNA total extraction for diagnostics of bacterial endosymbionts and DNA barcode methodology [20]. For *Culex* and *Anopheles* the morphological keys of Lane 1953 and Forattini 2002 [69, 70], and González & Carrejo 2007 [71] were used, respectively, to perform the recognition of sex and taxonomic identification. Two legs were collected for DNA barcode methodology and the rest of the body for diagnostics of bacterial endosymbionts [20]. All mosquitoes and phlebotomines were processed using a chill table at −5°C during the taxonomical identification process.

### DNA extraction from sand flies and mosquitoes for detection of endosymbionts

DNA extraction of all samples of sand flies and controls (Mosquitoes of *Aedes*) was performed according to the high salt concentration protocol [72]. The elution of total DNA was made in 50 μL of Buffer Tris-HCL 10 mM-EDTA 1 mM pH = 8. For *Anopheles* and *Culex* individuals, the body was processed with the ZR Tissues & Insect DNA mini-Prep (Zymo Research) extraction kit. The DNA concentration for the species of *Anopheles* and *Culex* varied approximately 45–280 ng/μl and for *Lutzomyia* species between 1 and 20 ng/μl on average measured using NanoDrop 2000 Spectrophotometer (Thermo Scientific NanoDrop, Wilmington, USA).

### Detection of bacterial endosymbionts of sand flies and mosquitoes by conventional PCR

*Wolbachia* detection in the collected insects was performed by PCR using *wsp*81F and *wsp*691R primers to amplify a partial fragment (650 bp) of the gene coding for the main surface protein of endosymbiotic *Wolbachia* (*wsp*) (Table 1) [73]. As a PCR-positive control, in all trials DNA from *Aedes* (*Stegomyia*) *aegypti* larvae from Medellín (Antioquia, Colombia) infected with a reference strain of *Wolbachia* (Supergroup A, strain *wMel*) were included, and ultrapure water was used as a PCR-negative control. *Microsporidia* detection was done using ss18f and ss1492r primers of the small subunit rDNA (ssrDNA- fragment 1300 bp) (Table 1) [37, 74]. A plasmid with *Microsporidia* MB (from *Anopheles arabiensis*) cloned fragment was routinely used as a positive control and ultrapure water as a PCR-negative control.

**Table 1:** Genes and primers used in polymerase chain reaction (PCR) assays to detect reproductive parasites in insect vectors (Phlebotominae Sand flies, *Culex*, *Anopheles*), DNA barcoding for the identification of species, screen of *Leishmania* infection and microbiota by Illumina MySeq from samples of several locations in Colombia.

Endosymbiont	Primer name	Primer sequence	PCR Cycle	Annealing Temp	Product Size (bp)	Reference
*Wolbachia*	wsp81F	F- 5’TGGTCCAATAAGTGATGAAGAAAC3’	35	58°C	610	74
	wSp691R	R- 5’AAAAATTAAACGCTACTCCA3’				
*Microsporidia*	ss18sf	F- 5’GTTGATTCTGCCTGACGT3’	35	45°C	1474	38, 75
	ss1492r	R- 5’GGTTACCTTGTTACGACTT3’				
*Cardinium*	CLOF1	F- 5’GGAACCTTACCTGGGCTAGAATGTATT3’	35	55°C	468	25
	CLOR2	R- 5’GCCACTGTCTTCAAGCTCTACCAAC3’				
*Spiroplasma*	spixo F	F- 5’TTAGGGGCTCAACCCCTAACC3’	35	52°C	810	38
	spixo R	R- 5’TCTGGCATTGCCAACTCTC3’				
COI	LCO1490	F- 5’GGTCAACAAATCATAAAGATATTGG3’	35	46°C	700	77,78
	HCO2198	F- 5’TAAACTTCAGGGTGACCAAAAAATCA3’				
HSP-70N	HSP70-F25	F- 5’GGACGCCGGCACGATTKCT3’	35	46°C	593	80
	HSP70-R617	F- 5’CGAAGAAGTCCGATACGAGGGA-3’				
Region V4 of the 16S rRNA	515F 806R	5’-GTGYCAGCMGCCGCGGTAA-3’ 5’-GGACTACNVGGGTWTCTAAT-3’	30	50°C	250	90

To detect *Cardinium*, we used the primers CLO-F1 and CLO-R1, targeting a ~450 bp fragment of the 16S *rRNA* gene (Table 1) [24]. As a positive control, *Cardinium* DNA from a biting midge (Culicoides) was included and ultrapure water as a negative control. Also, the presence of *Spiroplasma* was investigated using SpixoF and Spixo R primers that amplify a ~800 bp fragment of the *16S rRNA* gene (Table 1) [37]. As positive control, DNA of *S*. *poulsoni (SPHY1)* was included, and ultrapure water was used as a negative control. The reaction mix used and conditions of amplification for PCR for endosymbionts were done according to the conditions described previously [28, 37, 73, 74]. The amplified fragments were run on an agarose gel at 1% in 1× TBE buffer (40-mM Tris-Borate, 1 mM EDTA, pH 8.0) to verify the integrity of the fragments.

PCR products of each endosymbiont were ligated into a pJET1.2/blunt cloning vector following the manufacturer’s instructions (Thermo Fisher Scientific, MA, USA) [75]. Plasmidial DNA from a positive colony was extracted using GenJet Plasmid Miniprep Kit (Thermo Fisher Scientific, MA, USA), and amplified using pJET1.2 forward and reverse primers. Clones with the partial products of endosymbionts were verified by sequencing in both directions, using the service of Macrogen Company in Korea. For each assay, a negative control (no DNA), as well as a positive control (control PCR product by the cloning kit), was included. The taxonomic list of insect species, distribution, the frequency or percentage infection of endosymbionts by locality and ecoregion, as well as the sex ratio for each endosymbiont, was consolidated systematically.

### DNA barcoding identification of Phlebotominae sand flies and mosquitoes species

DNA samples of sand flies and mosquitoes positive for any endosymbionts and with taxonomic importance were used for PCR, corresponding to a barcode region of the COI (700 bp fragment) using LCO1490- HCO2198 primers (Table 1); the reaction mix, and conditions of amplifications were used according to the previously described [76, 77]. To amplify the mitochondrial 5′ COI gene region, PCR was performed using the following thermal profile: an initial denaturation of 5 min (94°C); followed by 35 cycles at 94°C for 1 min (denaturation), 45°C for 1 min (annealing), and 72°C for 1 min (extension); and a final extension at 72°C for 10 min. Each PCR cocktail had a final reaction volume of 50 μl and contained the following: 0.3 μL of Taq DNA polymerase (5 U/μL), 5 μL of 10× PCR buffer (NH4SO4), 5 μL of 2.5 mM MgCl2, 5 μL of 2 mmol/L dNTP, 2 μl of 10 μmol/L of each oligonucleotide (forward and reverse), 4 μL of the DNA template, and ddH2O to bring the volume to 50 μl [78]. The amplified fragments were observed on an agarose gel at 1% to verify the integrity of the fragments. Subsequently, double-stranded DNA was sequenced in both directions using a capillary electrophoresis system, using the service of Macrogen Company in Korea.

### PCR amplification of the HSP-70 N *Leishmania* gene in female groups

PCR was done to screen *Leishmania* infection in *Lutzomyia females*. HSP70-F25 and HSP70-R617 primers were used to amplify a 593 bp partial segment of the HSP-70N gene (coding for Heat shock protein 70) (Table 1). PCR was done following the described conditions by Fraga et al., 2010 [79]. As a positive control, *Leishmania panamensis* (reference strain UA140) DNA and *Leishmania braziliensis* (reference strain UA 2903) DNA were used.

### Molecular identification and phylogenetic analysis of endosymbionts based on the analysis of sequences

In this study we propose a genomic–phylogenetic species concept (GPSC) for the taxonomy of the S-symbionts [80]. The advantages of the GPSC are: *i)* The GPSC is a methodological species concept that relies heavily on macromolecular sequences (e.g. protein, RNA and DNA) that are not subject to biases of interpretation; *ii)* The organism’s ecology is encompassed by the GPSC in that adaptive radiations will bereflected in the alteration of gene and protein sequences in response to the organism’s niche; *iii)* The biogeography of an organism is considered by the GPSC in that genetic drift will result in a change in the gene and protein sequences of core genes; *iv)* The evolutionary history of the organism isconsidered through the phylogeny of gene and protein sequences; *v)* Sequence information is readily portable and globally accesible; *vi)* Taxa can be identified rapidly once the set ofrelevant genes for a have been identified.

Sequences of *wsp* gene and 16S rDNA obtained from *Wolbachia* or other endosymbionts, were directly compared with those in GenBank by BLASTn with megablast algorithm (https://blast.ncbi.nlm.nih.gov/Blast.cgi). The sequences were edited using Bioedit software v.7.2.5 [81]. Alignments of sequences of endosymbionts obtained and reported in GenBank were performed using a *Clustal W* incorporated in MEGA X software [82]. Verification of recombination events and the presence of chimeras was performed using RDP4 (Recombination Detection Program version 4) software [83]. Patterns of genetic divergence as nucleotide composition, the number of haplotypes (H) and variable sites were evaluated using Bioedit v.7.2.5(82) and DNAsp 5.0 [84]. K2P genetic distances of Kimura-2-Parameters (Intra and interspecific) also were evaluated with the MEGA X software [82]. The K2P model will be calculate the number of differences accumulated between two sequences since their last common ancestor. The K2P model is probably the most widely used of all models of nucleotide substitution for estimating genetic differences (called genetic distances) and phylogenetic relationships. The web program FINGERPRINT (http://evol.mcmaster.ca/fingerprint/) was also used to depict the nucleotide heterogeneity and nucleotide diversity [85].

Phylogenetic inference analysis for *Wolbachia*, *Cardinium*, and *Microsporidia* was performed using the Bayesian inference method with MrBayes 3.2 software [86] under the substitution model 4by4 (number of generations = 1’000.000), after the consensus trees were obtained, they were visualized and printed using FigTree V.1.1.4. We included a list of sequences previously registered for *Wolbachia* [20], *Cardinium* [22], *Microsporidia* [87] from arthropods for analysis. PCR products, and smaller edited sequences (< 550-600pb) were not considered in the phylogenetic analysis of *Wolbachia*. Alignments of sequences of *wsp* genes from sand flies and mosquitoes were performed as suggested Zhou *et al*., 1998 and Braig *et al*., 1998 [73, 88]. However, sequences of *wsp* gen less than 500 bp were also verified in BLASTn only to confirm the presence of *Wolbachia* and estimate infection rates. The phylogenetic relationship of *Microsporidia* included two fungi (*Basidiobolus ranarum* and *Conidobolus coronatus*) as outgroups. In the analysis of *Wolbachia* was used *Wolbachia* of *Bemicia tabaci* as outgroup. For phylogenetic inference of *Cardinium* was used as an outgroup *Candidatus Amoebophilus asiaticus*. The neighbor-joining (NJ) tree was built in MEGA X for analysis of sequences COI obtained to determine the limitation taxonomical for the identification of sand flies and mosquitoes.

### Interactions between the microbiota-endosymbionts: 16S rRNA gene survey, and statistics

To evaluate the dynamics of the microbiota against the presence or absence of different endosymbiotic bacteria, DNA of sand flies, and mosquitoes positive for one or more endosymbionts were considered (Table 2). Total DNA was used as a template to generate amplicons of the hypervariable region V4 of the 16S rRNA gene according to the protocol described at EMP 16S Illumina Amplicon Protocol (https://www.earthmicrobiome.org/protocols-and-standards/16s/) [89] using primer 515F and primer 806 R with PrimeSTAR HS DNA Polymerase (Takara, Japan) cocktail mix using the manufacturer’s instructions [90]. The first PCR round consisted of an initial step of 5 min at 95°C, followed by 20 cycles of denaturation at 98°C for 10 s, annealing at 50°C for 15s, and extension at 72°C for 45 s, followed by an elongation step of 72°C for four minutes and a holding stage at 4°C until further processing. From these reactions, 1 μl was transferred to fresh PCR cocktail mixes containing each corresponding overlapping primer with two distinct indices and Illumina adapters for each sample [91], run for 10 cycles using the same temperature and times of the first round. The presence and expected size of PCR products were assessed by agarose gel electrophoresis. Controls negative and positive were used.

**Table 2:** Samples of phlebotomine sand flies and mosquitoes that were positive for endosymbionts (Wolbachia*; Cardinium^, *Microsporidia*^*+*^) by PCR previously and selected for analysis of the total microbiota by Illumina MiSeq sequencing.

Species of sand flies or mosquitoes	Code	Department (Locality)	The concentration of DNA total (ng/ul)
Lutzomyia gomezi*	*LugomAnt120*	Antioquia (Puerto Triunfo)	21.8
Brumptomyia hamata*	*BrhamAnt117*	Antioquia (Puerto Triunfo)	68.2
Pintomyia evansi* ^	*PievaSuc11_15*	Sucre (Sabanas del Potrero)	5
Evandromyia dubitans*	*EvdubCor16_22*	Cordoba (Los Cordobas)	7.8
Evandromyia dubitans*	*EvdubCor51*	Cordoba (Valencia)	15
Psychodopygus panamensis*	*PspanCor4*	Cordoba (Montelibano)	14
Pintomyia evansi*	*PievaCor5*	Cordoba (Planeta Rica)	8.6
Micropigomyia micropyga*	*MimicCor257*	Cordoba (Planeta Rica)	1.79
Culex nigripalpus*	*CunigAnt73_95*	Antioquia (Puerto Triunfo)	30.2
Culex nigripalpus*	*CunigAnt96-101*	Antioquia (Puerto Triunfo)	45
*Pintomyia pia* ^***+***^	*PipiaCal139*	Caldas (Salamina)	143.9
*Pintomyia evansi* ^***+***^	*PievaCor17*	Cordoba (Planeta Rica)	3
*Culex nigripalpus* ^***+***^	*CunigAnt84*	Antioquia (Puerto Triunfo)	5.8

Amplified products were purified, normalized, and pooled using the SequalPrep Normalization Plate (Thermofisher Scientific, USA) and subjected to 250-bp paired-end Illumina MiSeq sequencing, using MiSeq Reagent Kit, (300-cycles). On negative and positive controls of sequencing, in this case they were not necessary since there were no negative control amplification signals and the sequencing kit produced sequences of high- quality and expected quantities, as it can be confirmed inspecting the raw data quality values and reads per sample. Sequencing was commissioned to Alianza Sanitizar SA. From demultiplexed 16S amplicon raw pair-end sequence datasets from each sample, we used the DADA2 software package (https://github.com/benjjneb/dada2) following a sequential pipeline for filtering, denoising, chimeras, and merging [92], resulting in assembled datasets for each amplicon with trimmed reads based on quality, cut out of the primer sequences, deleting potentially chimeric sequences, to detect the counts of each unique Amplicon Sequence Variant (ASV) across all samples and to classify them using RDP Naive Bayesian Classifier [93] having as taxonomic reference Silva database release 132 (https://www.arb-silva.de/documentation/release-132/).

In all cases, the coverage was above >0.9 with 10.000 reads, therefore Bray-Curtis dissimilarity matrix of non-rarified relative percentages was used. For Bray-Curtis dissimilarity between all pairs of samples at ASV level was calculated using phyloseq (default parameters, https://joey711.github.io/phyloseq/distance.html). Identical nodes on displayed UPGMA dendrogram clustering were obtained with data rarified to 19884 reads on each dataset. The phyloseq software package (https://joey711.github.io/phyloseq/) [94] and Microbiome Analyst (https://www.microbiomeanalyst.ca) [95], were used for estimating and obtain plot on alpha diversity (within-sample) metrics (Observed, Chao 1, Shannon, Simpson) and beta diversity between all communities (Hierarchical clustering analysis, Principal Coordinates Analysis of weighted Unifrac distances-PCoA, Heatmap). Non-metric Multidimensional Scaling (NMDS) was also obtained with Microbiome Analyst using Bray-Curtis index [95]. Differences in weighted and unweighted Unifrac distances between the groups of sand flies and mosquitoes were analyzed with analysis of similarity (Kruskal-Wallis test). A P value ≤0.05 was considered statistically significant. The core microbiota (Sample prevalence 20%; Relative abundance 0.01%) and linear discriminant effect size (LEfSe) analysis were implemented using Microbiome Analyst [95], to identify that were significantly and differentially abundant taxa between hosts. A threshold alpha value of 0.05 for the LSmeans & Tukey’s HSD post-hoc test was implemented. The random forest algorithm was also used to find significant correlations between bacterial taxa using the top 25 most abundant genera of the microbiota.

## Results

### Diversity of sand flies and mosquitoes

Five hundred seventy-four individuals (292 and 282 from the Caribbean the Andean region, respectively) were collected and identified by classical taxonomy. 430 specimens from 12 genera of phlebotomines, 109 individuals from seven species of the genus *Anopheles* and 35 individuals corresponding to *Culex nigripalpus* (Table 3). Species with abundances greater than 50 specimens correspond to *Pi*. *evansi*, *Lu*. *longipalpis* and *An*. *nuneztovari* (Table 3).

**Table 3:** List of species of phlebotomine sand flies, *Culex nigripalpus* and *Anopheles* tested for *Wolbachia*, *Cardinium*, Microsporidia and *Spiroplasma* in two ecoregions from Colombia.

	Andean region										Caribbean region			*Endosymbionts for species*				
Departments	Cordoba								Sucre	Caldas	Antioquia	Cundinamarca						
Municipality or province	Los Córdobas	Valencia	Tierralta	Pto. Libertador	Montelíbano	Planeta Rica	Monteria	Sahagún	Sabanas del Potrero	Salamina	Puerto Triunfo	Ricaurte	Total	*Positive individual for Wolbachia (%)*	*Positive individuals for Microsporidia (%)*	*Positive individuals for Cardinium (%)*	*Positive individuals for Spiroplasma (%)*	*Dual infected*
**Sand flies and mosquito species**																		
*Micropygomyia atroclavata*	*-*	1	-	-	-	-	-	1	-	-	-	-	2					
*Psathyromyia carpenteri*	*-*	1	-	-	1		-	-	-	-	10	-	12					
*Micropygomyia cayennensis* ^***+***^ ^*	5	-	-	8	4	1	-	-	-	-	6	-	24	**1(4,1)**		**11 (45,83)**		
*Evandromyia dubitans* ^***+***^	3	1	1	-	-	-	-	-	-	-	-	-	5	**5(100)**				
*Pressatia dysponeta*	*-*	-	-	3	-	-	-	-	-	-	-	-	3					
*Pintomyia evansi* ^***+#***^ ***^***	4	1	9	9	4	13	-	-	40	-	-	-	80	**9 (11,3)**	**2(2,5)**	**1(1,15)**		**1(1,25)**
*Lutzomyia gomezi* ***^***	*-*	3	-	10	-	11	-	12	-	-	4	-	40	**2(5)**				
Micropygomyia micropyga*	*-*	-	-	-	-	1	-	-	-	-	-	-	1	**1(100)**				
*Pintomyia rangeliana*	2	2	-	-	2	-	1	1	-	-	-	-	8					
*Psychodopygus panamensis* ^*	*-*	2	9	-	5	2	-	-	-	-	8	-	26	**1(3,8)**				
Psathyromyia shannoni*	*-*	-	-	-	-	-	9	1	-	-	2	-	12			**3(25)**		
*Luztomyia longipalpis*°***^***	*-*	-	-	-	-	-	-	-	-	-	-	75	75					
*Micropygomyia trinidadensis* ***^***	*-*	-	-	-	-	-	-	-	-	-	35	-	35					
Brumptomyia hamata*	*-*	-	-	-	-	-	-	-	-	-	6	-	6	**1(16,6)**				
*Pressatia camposi*	*-*	-	-	-	-	-	-	-	-	-	8	-	8					
*Dampfomyia vespertilionis* ^***+***^	*-*	-	-	-	-	-	-	-	-	-	4	-	4					
*Nyssomyia trapidoi* ***^***	*-*	-	-	-	-	-	-	-	-	-	4	-	4					
*Nyssomyia yuilli yuilli* ***^***	*-*	-	-	-	-	-	-	-	-	-	10	-	10					
*Evandromyia walkeri*	*-*	-	-	-	-	-	-	-	-	-	2	-	2					
Trichopygomyia triramula*	*-*	-	-	-	-	-	-	-	-	-	2	-	2			**1(50)**		
*Pintomyia ovallesi*	*-*	-	-	-	-	-	-	-	-	-	4	-	4					
Pintomyia pia*	*-*	-	-	-	-	-	-	-	-	48	-	-	48		**1(2,1)**			
*Pintomyia columbiana* ***^***	*-*	-	-	-	-	-	-	-	-	13	-	-	13					
*Pintomyia nuneztovari*	*-*	-	-	-	-	-	-	-	-	5	-	-	5					
*Pifanomyia longiflocosa* ***^***	*-*	-	-	-	-	-	-	-	-	1	-	-	1					
*Anopheles nuñeztovari* ***^***	19	29	16	-	-	-	-	-	-	-	-	-	64					
Anopheles trianulatus*	*-*	-	-	8	-	-	-	4	-	-	-	-	12	**2 (16,6)**				
*Anopheles albimanus* ***^***	15	-	1	-	2	6	-	-	-	-	-	-	24					
*Anopheles darlingi* ***^***	*-*	-	-	-	1	1	-	-	-	-	-	-	2					
*Anopheles oswaldoi* ***^***	*-*	-	-	-	5	-	-	-	-	-	-	-	5					
*Anopheles cf*. *Janconnae*	*-*	-	-	-	-	1	-	-	-	-	-	-	1					
*Anopheles pseudopuctipennis*	*-*	-	-	-	-	1	-	-	-	-	-	-	1					
*Culex nigripalpus* ^Ω***+***^ ***^***	*-*	-	-	-	-	-	-	-	-	-	35	-	35	**7 (20)**	**1 (2,8)**			
**Total (%)**	**48**	**40**	**36**	**38**	**24**	**37**	**10**	**19**	**40**	**67**	**140**	**75**	**574**	**29 (5,05)**	**4 (0,7)**	**15 (2,6)**	**0(0)**	**1(1,25)**
*N* (***% Wb Sand flies—% Mosquitoes***)	**183 (9,83**) - **109 (1,83)**									**247 (2,83) - 35 (20)**								
*N* (***% Mp Sand flies****—****% Mosquitoes***)	**183 (1,1) - 109 (0)**									**247 (0,8) - 35 (2,8)**								
*N* (***% Cd Sand flies—% Mosquitoes***)	**183(8,74)** - **109 (0)**									**247 (0,4)** - **35 (0)**								
*N* (***% Sp f Sand flies—% Mosquitoes***)	**183 (0) - 109 (0)**									**247 (0)** - **35 (0)**								

N: Number of individual. %: Percentage.

Wb: Wolbachia. Mp: Microsporidia. Cd: Cardinium. Sp: Spiroplasma

Reported with Wolbachia^+^, Microsporidia°, Cardinium^#^, Spiroplasma^Ω^, New report with endosimbiont*. Medical importance^

### Frequency and molecular detection of endosymbionts

Conventional PCR used in this study could detect *Wolbachia*, *Cardinium*, and Microsporidia in total DNA extracted from sand flies and mosquitoes using reference primers (S1 Fig). Only *Wolbachia wsp* primers showed some PCR products of unexpected size (S1C Fig. Line 11). *Spiroplasma* was not detected in the DNA samples using reference conditions. Correct cloning of PCR products from various endosymbionts from natural populations of sand flies and mosquitoes from Colombia was successfully performed and preserved (S1H Fig). In the Caribbean region, *Wolbachia* was detected in 9.83% of the sand flies, and 1.83% of the mosquitoes were positive (Table 3).

In contrast, lower *Wolbachia* infection values were found in sand flies (2.83%) and higher in mosquitoes (20%) in the Andean region (Table 3). The presence of *Microsporidia* in the Andean region (mosquitoes 2.8%, sand flies 0.8%) and Caribbean ecoregion (sand flies 1,1%) was low (Table 3. *Cardinium* was estimated with lower infection values in the Andean region (sand flies 0.4%) but with a significant presence in sand flies (8.74%) from the Caribbean ecoregion (Table 3).

The results by the department show the highest presence of *Wolbachia* in Sucre (11%) compared with Antioquia (1%) and the Córdoba (5%) (Fig 1). For *Cardinium*, similar infection rates (5%) for the Córdoba and lower levels in Sucre (2%) and Antioquia (1%) (Fig 1). Natural populations of sand flies and mosquitoes from Antioquia and Caldas showed lower levels of *Microsporidia* infection (1%) compared to Sucre (4%) (Fig 1).

Specific analyses show that *Wolbachia* was found in seven species of sand flies and two species of mosquitoes (Table 3). A high percentage of infection was observed in *Ev*. *dubitans* (100%), *Pi*. *evansi* (11.3%) and *Br*. *hamata* (16.6%). Importantly, a single diagnosed *Mi*. *micropyga* individual was infected with *Wolbachia*. The mosquitoes, *Cx*. *nigripalpus* (20%) and *An*. *trianulatus* (16.6%) also presented high levels of infection (Table 3). Species such as *Pi*. *pia* (2.1%), *Pi*. *evansi* (2.5%) and *Cu*. *nigripalpus* (2.8%) were the only ones that showed positive results for *Microsporidia*. The species *Mi*. *cayennensis* (population from the department of the Córdoba) presented a high percentage of *Cardinium* infection (45.83%) followed by *Tr*. *triramula* (50%) and *Ps*. *shannoni* (25%) (Table 2). Double infection with *Wolbachia* and *Cardinium* is reported in one *Pi*. *evansi* specimen from the Sucre department (S2 Table and Fig 1C and 1F). For the first time, *Wolbachia*, *Cardinium*, and *Microsporidia* endosymbionts are registered in different species of sand flies and mosquitoes (Table 3).

We observed a higher frequency of *Wolbachia* presence in female sand flies and mosquitoes (9.27%) (Table 4). Contrasting *Cardinium*, was found only in phlebotomines and this has a more equitable sex ratio of infection (Males = 3.43%; Females = 2.41%) (Table 4). *Microsporidia* was detected only in females (1.03%) (Table 4).

**Table 4:** The frequency of endosymbionts infection by sex in sand fly and mosquitoes from Colombia.

	Sex	Number of females positive			Number of males positive	
Sand flies and mosquitoes species	Number total of individuals	*Wolbachia*	*Cardinium*	Microsporidia	*Wolbachia*	*Cardinum*
*Pi*. *evansi*	80	8	1	1	1	1
*Ps*. *panamensis*	26	1		-	-	-
*Mi*. *cayennensis*	24	1	4	-	-	7
*Lu*. *gomezi*	40	2		-	-	-
*Ev*. *dubitans*	5	4		-	1	-
*Mi*. *micropyga*	1	1		-	-	-
*Ps*. *shannoni*	12	-	1	-	-	2
*Pi*. *pia*	48	-		1	-	-
*Tr*. *triramula*	2	-	1	-	-	-
*Br*. *hamata*	6	1	-	-	-	-
*An*. *trianulatus*	12	2	-	-	-	-
*Cx*. *nigripalpus*	35	7	-	1	-	-
***Total (%)***	**291**	**27 (9,27)**	**7 (2,41)**	**3 (1,03)**	**2 (0,68)**	**10 (3,43)**

### Molecular identification and phylogenetic relationships of endosymbionts

**Fig 2:**
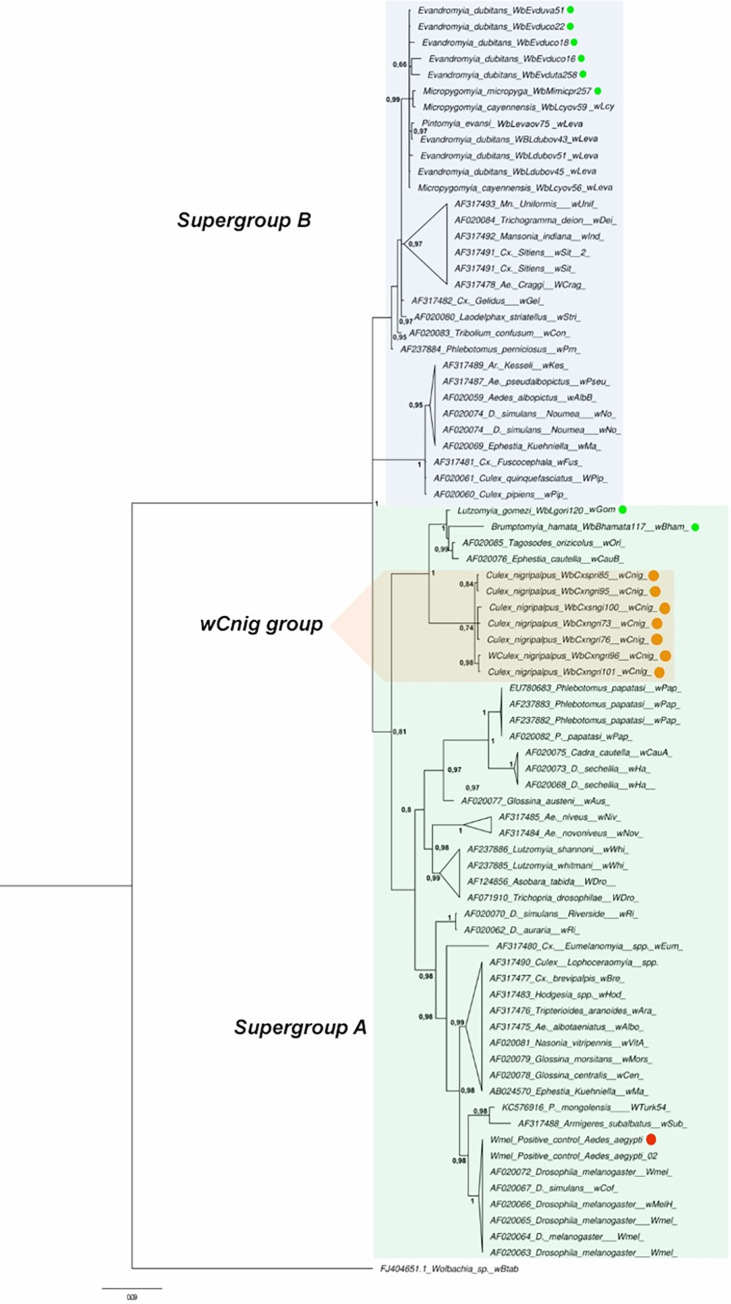
Phylogenetic relationships of *Wolbachia* strains using Bayesian analysis and inferred with *wsp* sequences. The *Wolbachia* strains are identified by host species from which they were isolated (sand fllies symbol of green color; *Culex* symbol of orange color). Numbers in nodes represent Bayesian posterior probabilities. Reconstruction performed with MrBayes (version 3.0). The *wMel* positive control is indicated in red color. As out-group *Wolbachia* of *Bemicia tabaci*.

Fifteen *Wolbachia wsp* gene sequences (eight from phlebotomines sand flies; seven from *Culex nigripalpus*) presented high percentages of similarity (98% - 99%) compared with the GenBank sequences. The global nucleotide alignment (580 bp) of the *wsp* gene included an additional 63 sequences from 59 arthropod-derived *Wolbachia* strains located in super groups A and B, as seen in the phylogenetic relationships of Bayesian inference (Fig 2). Clade posterior probability ranged from 0.8 to 1, indicating a robust analysis of *Wolbachia* phylogeny (Fig 2). Host *wsp* sequences were determined for *Cx*. *nigripalpus*, *Br*. *hamata*, and *Lu*. *gomezi* from Antioquia (Andean Eco-region), located in supergroup A, while the *wsp* sequences of phlebotomines (*Mi*. *micropyga* and *Ev*. *dubitans)* from the Department of Cordoba (Eco-region Caribe) present a close relationship with strains of the "Leva" group located in supergroup B (Fig 2).

Seven *wsp* sequences obtained from *Cx*. *nigripalpus*, with all similar nucleotide sequence suggesting a new strain named *wCnig*, forming a putative new group Cnig (Fig 2). Cnig’s specific alignment (514 bp) suggests the presence of three haplotypes, 10 variant sites (S2 Fig), eight parsimoniously informative sites, and two singletons. Heterogeneity is distinguished at the beginning and end of the alignment (S2 Fig), indicating that K2P genetic distances ranged from 0.001 to 0.002 and the identity percentages between 98.33% and 100%. It was also determined that *Wolbachia wsp* gene sequences were obtained from *Lu*. *gomezi* corresponds to a variant or haplotype of the *wOri* strain, while *Br*. *hamata* corresponds to a putative new strain named wBham, which is also related to the *wOri* and *wCauB* strains of *Tosogodes* and *Ephetesia*, respectively (Fig 2). To confirm this result, 33 variable sites and three parsimoniously informative sites with a high number of singletons were determined in the specific alignment of this sequence grouping. The K2P genetic distances of *wLgom* with *wOri* and *wCauB* were 0.02, with *wCnig* 0.12 and 0.007 with *wBham*. The percentage of similarity of *wLgom* with *wOri* was 98.57%. In contrast, *wBham* presented distances with values of 0.17 with *wCnig* and 0.07 with *wOri* and *wCauB*. The percentage of similarity of *wBham* with *wOri* was 88.84%, as with *wLgom*, indicating a clear difference between these two strains.

*Wsp* sequences of *Mi*. *micropyga* and *Ev*. *dubitans* were consistently located in the Leva group, as mentioned above, indicating that nucleotide alignment revealed the existence of 29 variant sites, eight parsimoniously informative sites, and heterogeneity was appreciated throughout the alignment (S2 Fig). The K2P genetic distance values ranged from 0.001 to 0.003, indicating that the *Ev*. *dubitans* are similar to the *wLeva* strain (identity percentages 99.28% −98.8%), while the *Mi*. *micropyga* sequences are similar to the *wLcy* strain, where it presented identity percentages of 99.52%. The percentages of identity between sequences of *Mi*. *micropyga* and *Ev*. *dubitans* were 96.63% −98%.

Regarding *Microsporidia*, good quality 16S SSU rDNA sequences were obtained from PCR products detected in *Pi*. *pia* and *Cx*. *nigripalpus* with high percentages of similarity (99%) associated with *Microsporidia* sequences deposited in GenBank. The 16S SSU rDNA sequences from our study and 82 *Microsporidia* representing the five established clades derived from arthropods with different environmental origins were successfully aligned to achieve a data set of 1097 bp.

**Fig 3:**
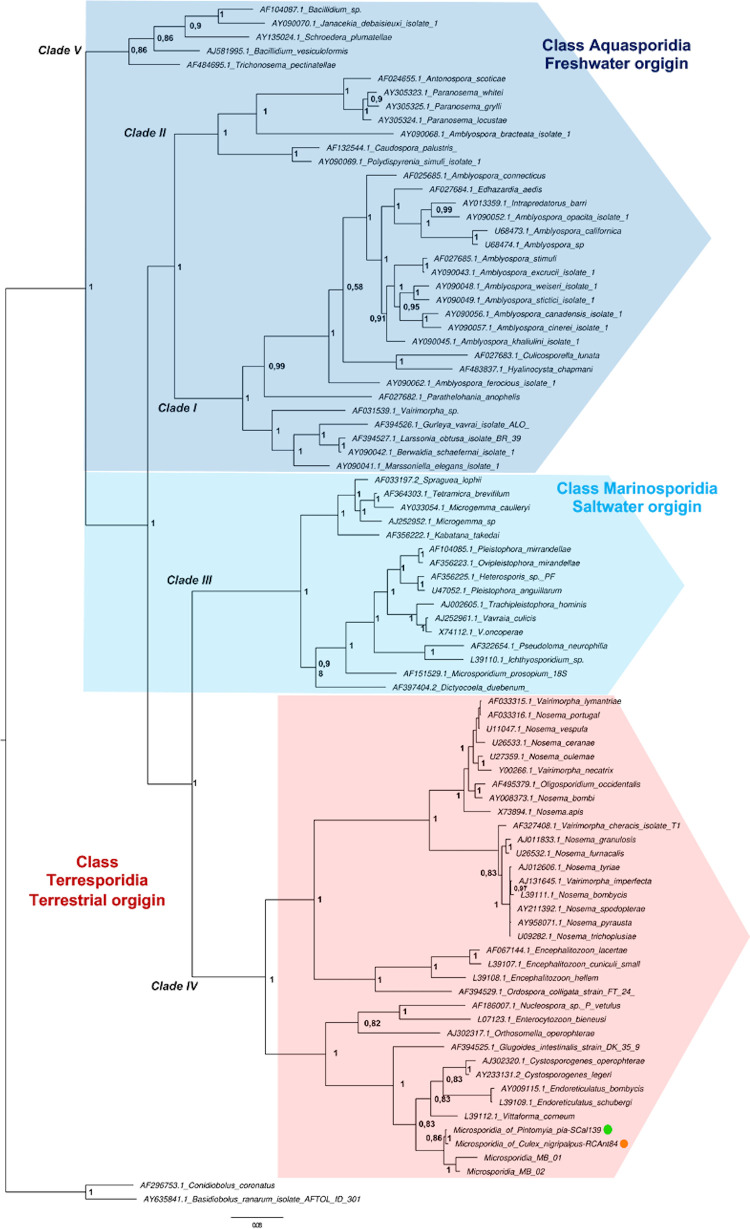
Bayesian inference and phylogenetic relationships of *Microsporidian* strains using sequences of the small subunit ribosomal DNA. The *Microsporidian* strains are identified by host species from which they were isolated (sand fly symbol of green color; *Culex nigripalpus* of orange color). Numbers in nodes represent Bayesian posterior probabilities. Reconstruction performed with MrBayes (version 3.0). Clades I–V are indicated. Two fungi were used as outgroups in the analysis (*Conidiobolus coronatos* and *Basidiobolus ranarun*). Taxa without GenBank accession numbers are original for this dataset.

The phylogenetic relationships determined by Bayesian inference (Fig 3) suggest that *Microsporidia* of *Pi*. *pia* and *Cx*. *nigripalpus* was located in clade IV corresponding to the Terresporidia class (Terrestrial Origin) and closely related to *Microsporidian* MB from *An*. *arabiensis* (ID 2270798) with supports of the posterior probability of 0.86 (Fig 3). Other nearby *Microsporidia* correspond to *Vitaforma*, *Endoreticulatus*, and *Cystoporogenes* (Fig 3). The analysis of genetic variability that includes sequences from this grouping (Clade) indicated the existence of 296 variant sites, with heterogeneity distributed throughout the alignment (S2 Fig). Contrasting, the 16S SSU rDNA sequences of *Microsporidia* MB, *Pi*. *pia* and *Cx*. *nigripalpus*, they revealed only 62 variants and 16 parsimoniously informative sites, showing clear differences. The K2P distances show values of 0.002 and 99.46% identity percentages between *Microsporidia* of *Pi*. *Pia* and *Cx*. *nigripalpus* indicating that they corresponded to the same species, but different from *Microsporidia* MB because the K2P genetic distances were 0.056 with identity percentages between 94.40% and 97.20%. Concerning the other members of nearby *Microsporidia*, the minimum values of K2P distances were 0.09 with *Vitaforma corneae* and maximums of 0.167 with *Endiriculatus bombicis*.

**Fig 4:**
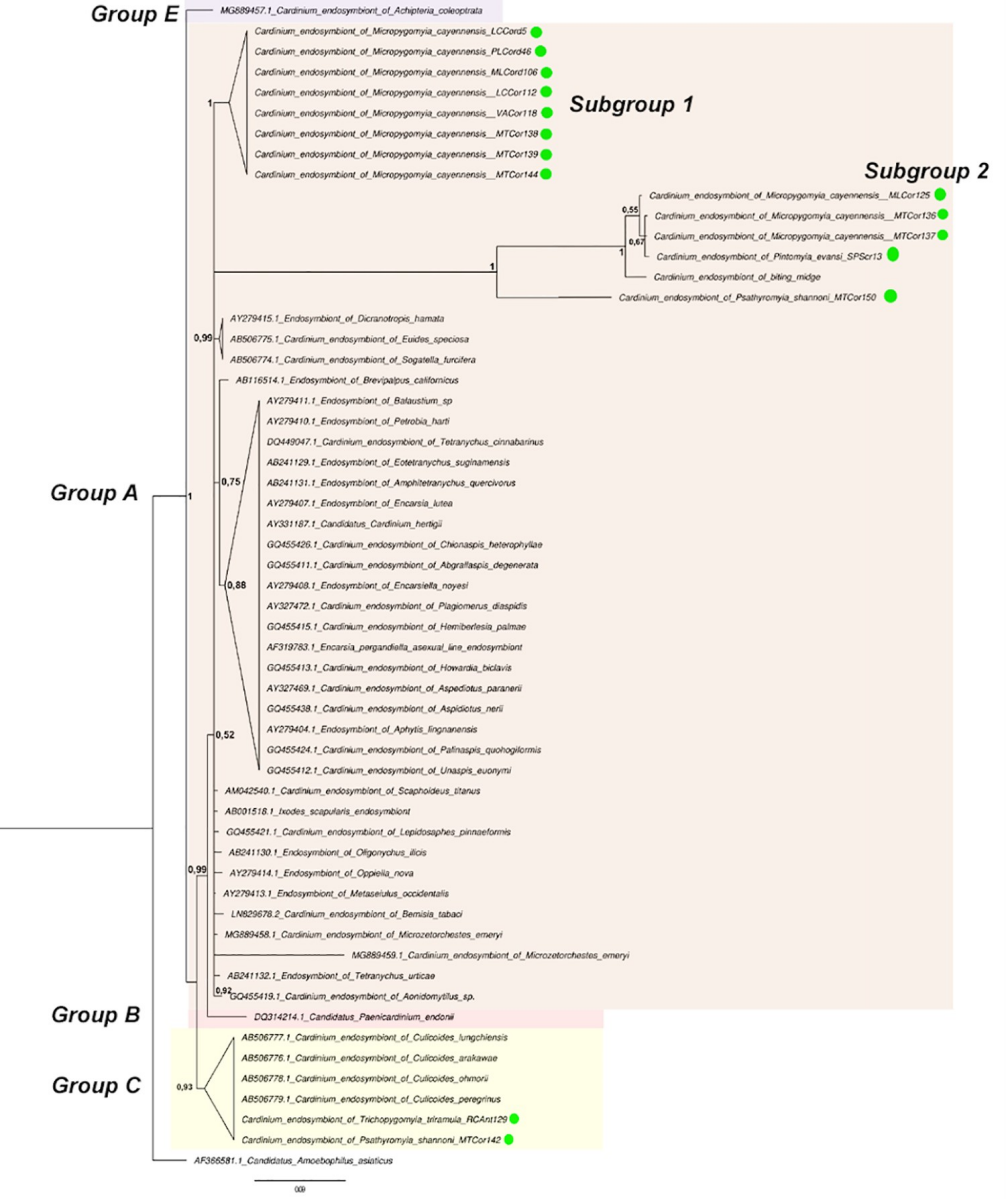
Phylogenetic relationships of *Cardinium* strains using Bayesian analysis and based on 16S rDNA sequences. The *Cardinium* strains are identified by host species from which they were isolated (sand flies symbol of green color). Numbers in nodes represent Bayesian posterior probabilities. The reconstruction was performed using MrBayes (version 3.0). As an outgroup was used *Candidatus Amoebophilus asiaticus*. Taxa without GenBank accession numbers are original for this data set.

The initial analysis of partial 16S rDNA sequences of *Cardinium* found in phlebotomines revealed high and variable percentages of similarity (97.61% −98.49%) with the arthropod host sequences reported in Genbank. In total 14 only phlebotomine sequences were obtained and compared with 41 available *Cardinium* sequences and located in groups A, B, C, and E (Fig 4), the result of a nucleotide alignment of 467 bp, of which 368 sites were variables. Specifically, the sequences are categorized as the *Cardinium* of *Tr*. *triramula* and *Ps*. *shannoni*, they were located in group C with a high posterior probability of clade (0.93), indicating a close relationship with the *Cardinium* of *Culicoides* (Fig 4). In this group C data set, 13 variant sites (S2 Fig) and four parsimoniously informative sites were estimated, with K2P genetic distances ranging between 0.001 and 0.002, and identity percentages between 97.67% and 98.19%, suggesting which corresponded to the same *Cardinium* species, but in different hosts. Heterogeneity occurred mainly at the beginning of the alignment (S2 Fig).

Two subgroups of *Cardinium* sequences, the first only associated with *Mi*. *cayennensis* (Subgroup 1, the posterior probability of clade = 1) and the second drift of *Mi*. *cayennensis* and *Pi*. *evansi* (Subgroup 2, the posterior probability of clade = 1) and one sequence near *Ps*. *shannoni* were located phylogenetically in group A (Fig 4). Further analysis suggests the presence of 17 variant sites in subgroup 1 and a single parsimoniously informative site (S2 Fig), while K2P distances were 0.001 between all sequences in this subgroup confirming that they corresponded to a single new strain called *Cardinium* of *Mi*. *cayennensis* sub-1. In subgroup 2, only three variable sites were determined, and the genetic distances ranged from 0.003 to 0.009, with the sequence of *Mi*. *cayennensis* MtCor125 is the most divergent. These results confirmed the presence of another genetic variant called the *Cardinium* of *Mi*. *cayennensis* sub-2. Our results contribute to the significant presence of *Cardinium* diversity in phlebotomine sand flies.

### DNA barcoding for the identification of Sand flies and mosquitoes’ species

**Fig 5:**
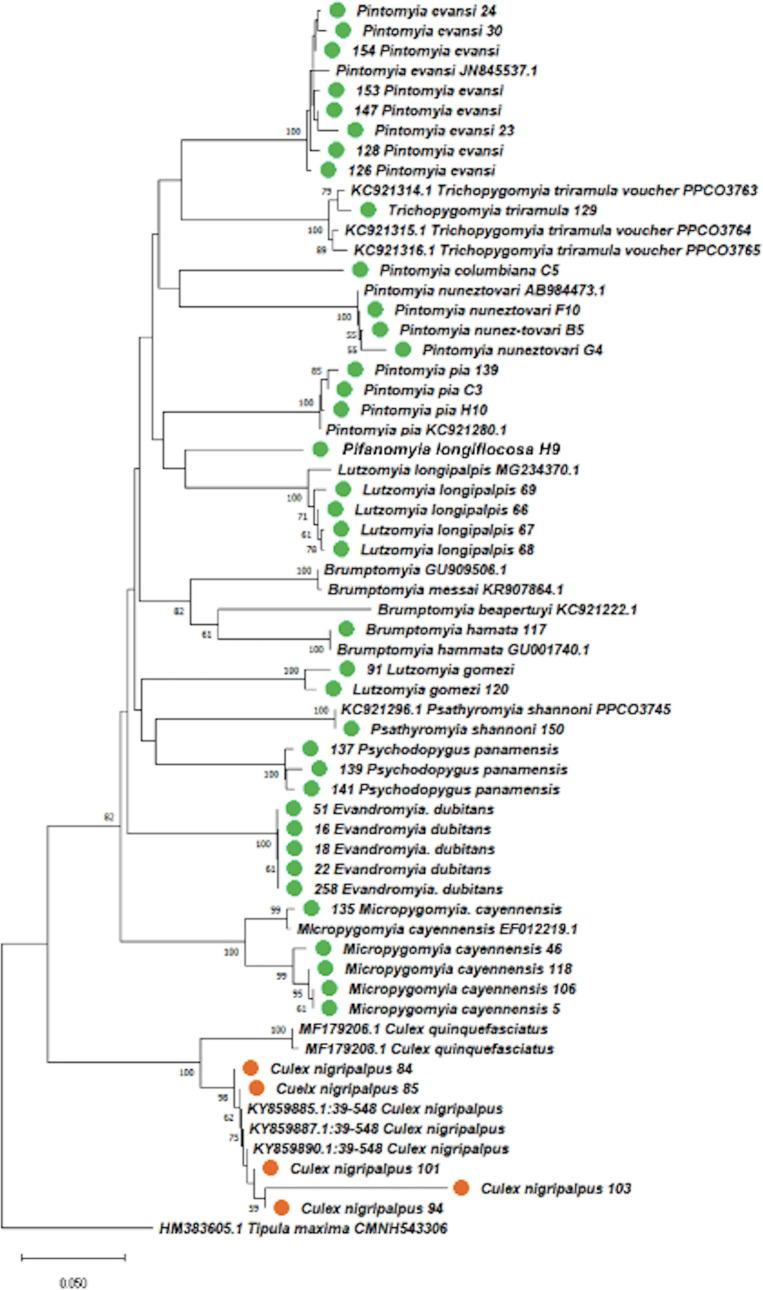
Neighbor-joining analysis of mitochondrial COI sequences among sand flies species (symbol of green color) and mosquitoes of genus *Culex* (symbol of orange color) from Colombia, based on the model of Kimura’s two-parameter model (K2P). Bootstrap support was estimated with 1000 replicas, only bootstrap values >50% being shown above the branch. As an outgroup was used *Tipula maxima*.

A total of 38 sequences barcode (COI gene) of phlebotomines and five sequences of mosquitoes positive in the diagnosis of endosymbionts and other species with difficulties in taxonomic determination by morphology were correctly analyzed and allowed species differentiation. Other sequences available at Genbank for phlebotomines from Colombia were included in the Neighbor-Joining comparative analysis (Fig 5). The analysis showed high bootstrap values (100%) for taxonomic groupings of sand flies species and *Cx*. *nigripalpus* (Fig 5).

### Interactions between the microbiota-endosymbionts in sand flies and mosquitoes

A total of 11 samples of the DNA of phlebotomines (n = 8) and *Cx*. *nigripalpus* (n = 3) that were positive for *Wolbachia*, *Microsporidia*, and *Cardinium*, were selected to evaluate the microbiota behavior by amplifying the V4 region of the 16S rRNA (Table 4).

The summary of results obtained (total reads, the number of ASVs, top five Phyla-Family-Genus) from 16S rRNA gene amplicon sequencing of 11 samples of insects, untreated and treated based on quality and taxonomy classification, is shown in the S1 Table and S3 Fig.

At the phylum level, the microbiota is dominated by five bacterial groups: Proteobacteria (67.6%), Firmicutes (17.9%), Actinobacteria (7.4%), Bacteroidetes (1.7%) and Cyanobacteria (1.5%), also with a 3,25% fungal phyla Microsporidia, making the 99.4% of the entire population (S4 Fig).

**Fig 6:**
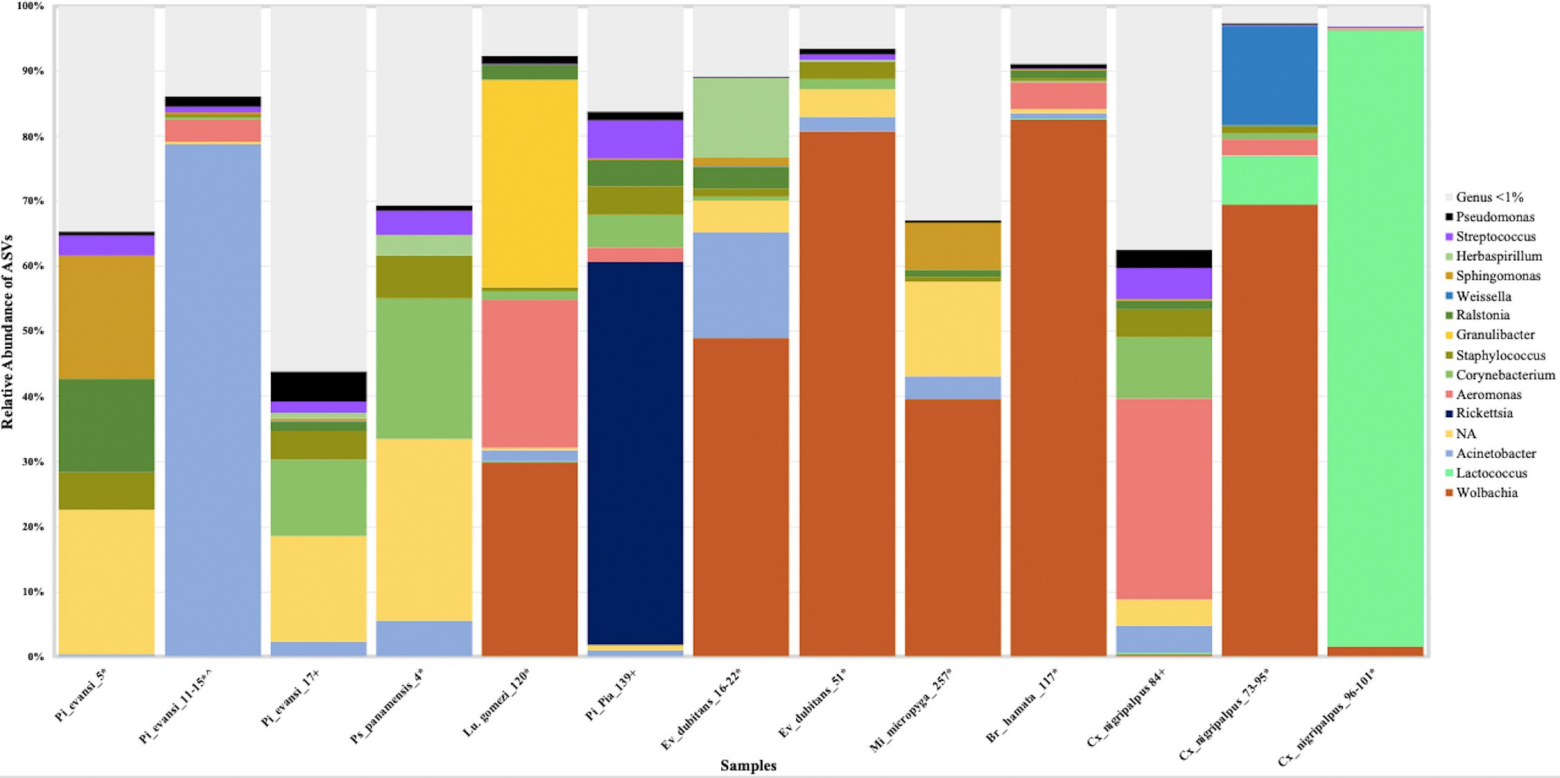
Microbiota composition and diversity of wild specimens of sand flies and *Culex nigripalpus* found with endosymbiont in several regions or departments from Colombia. A) Relative abundance of ASVs that were called to the taxonomic rank of genus. The ASVs with abundances above 1% of the rarefied count matrix were selected. See Table 4 for the nomenclature of samples in detail. NA: ASVs without taxonomic assignment.

The total microbiota profiles at the level of genus show high percentages of infection by *Wolbachia* in *Lu*. *gomezi*, *Ev*. *dubitans*, *Mi*. *micropyga*, *Br*. *hamata*, and *Cx*. *nigripalpus*, with relative abundances of ASVs of 30%-83% (Fig 6). A high significance in public health represents the first record of the natural infection of *Rickettsia* ASVs in sand flies from Colombia, mainly in populations of *Pi*. *pia* (Department of Caldas) with a relative abundance of 58.8% (Fig 6).

**Fig 7:**
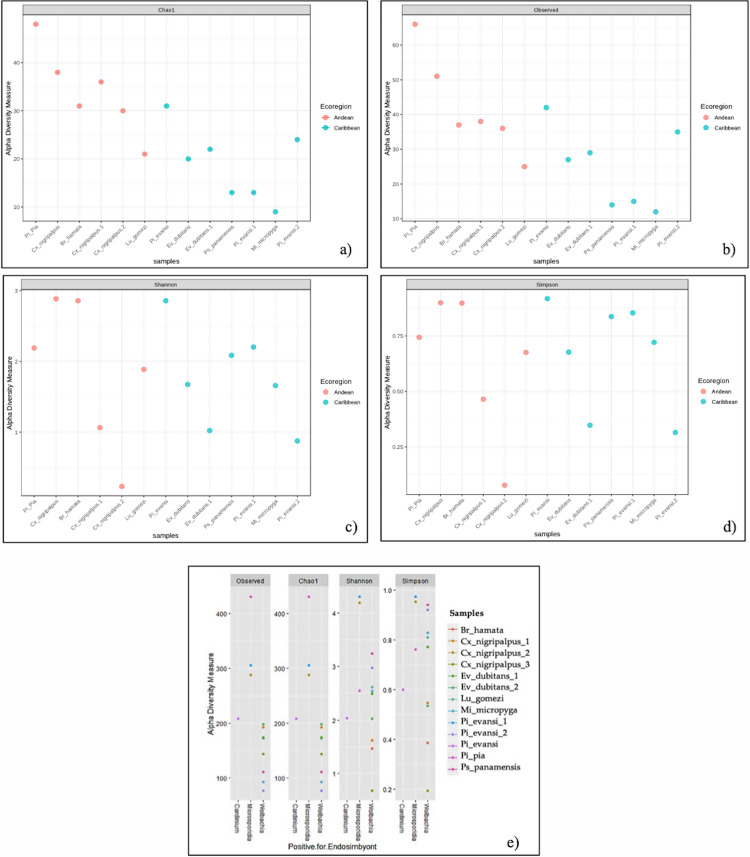
Alpha diversity (intra-diversity) of bacterial composition using amplicon sequence variants (ASVs) in sand flies and *Cx*. *nigripalpus* from Colombia by ecoregion and presence of endosymbiont. a) Species richness was assessed by Chao1 (estimates diversity from abundance data), b) Observed OTU (sum of unique OTUs in each sample), c) Shannon-Wiener (sum of species proportion logarithms) and d) Simpson indexes. e) Alpha diversity with all indexes but associated with the presence of endosymbionts. Higher index values indicate richer and more diverse bacterial composition.

**Fig 8:**
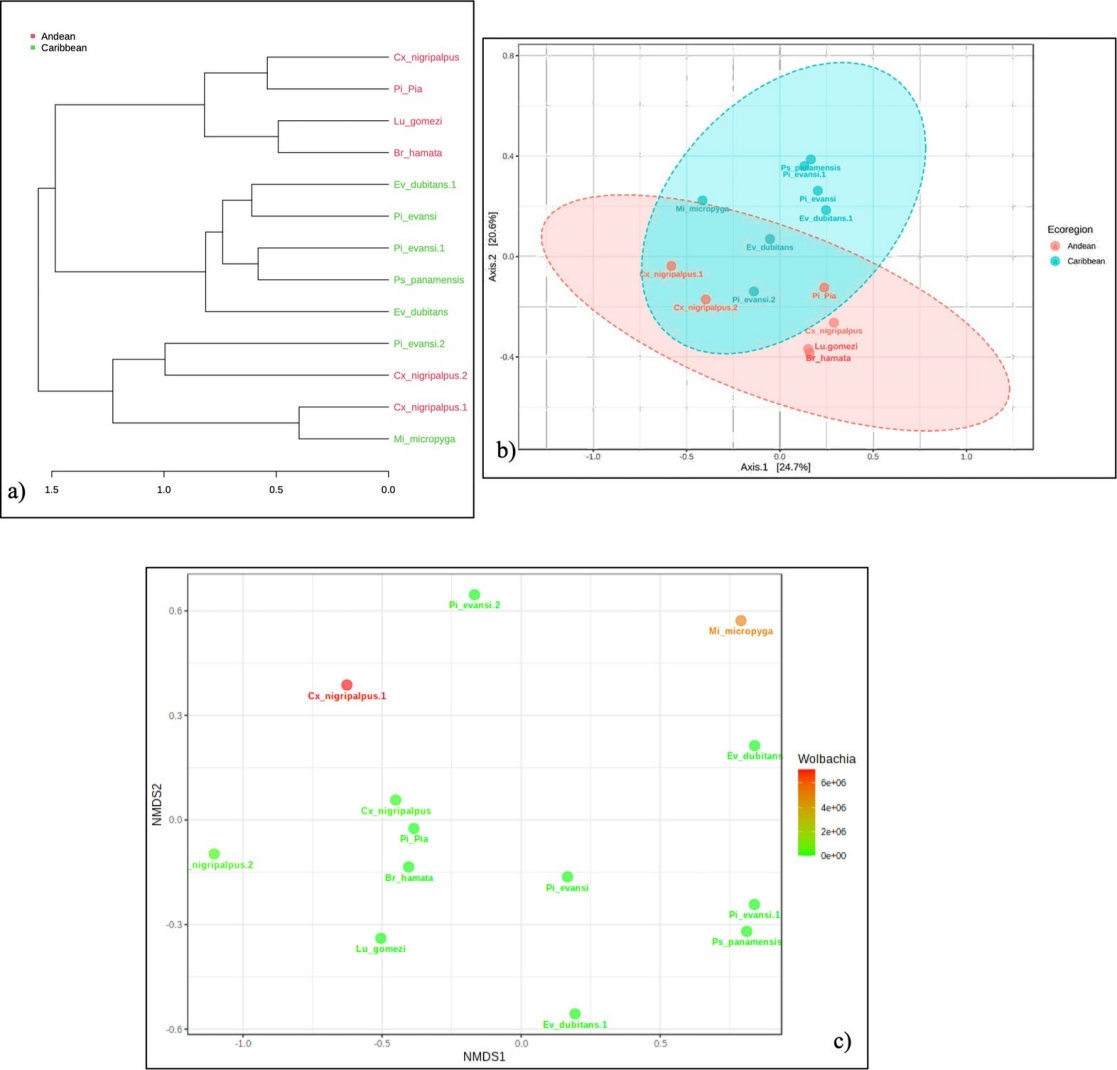
β-diversity analysis (between groups) of microbial communities associated with the groups of samples established of sand flies and *Culex nigripalpus* found with endosymbiont in several ecoregions from Colombia. a) Hierarchical clustering analysis of ASVs at the genus level. b) Principal Coordinate Analysis (PCoA) with ASVs at the genus level (Weighted Unifrac Distance, PERMANOVA, F = 2.4744; R2 = 0.18363; p-value = 0.007). c) Beta-diversity visualized using Non-metric Multidimensional Scaling (NMDS). The plot was obtained with Bray-Curtis Dissimilarity distances (PERMANOVA, F = 1.1429; R2 = 0.094118; p-value = 0.324; [NMDS] Stress = 0.11348).

In summary, there are clear differences in the composition and diversity of microbiota at the intra-specific (Figs 6 and 7) and interspecific (Fig 8) levels in sand flies and *Cx*. *nigripalpus*, which may depend in the first instance on the presence and load of natural infection of endosymbionts (Fig 6). For example, *Pi*. *evansi*, revealed two samples positive with *Wolbachia* and one with *Microsporidia* by PCR, but have a low relative abundance of these ASVs (0.04%-0,2%) in their microbiota, with differences in the composition of ASVs such as *Acinetobacter*, *Streptococcus*, *Staphylococcus*, *Ralstonia*, *Pseudomonas* and *Aeromonas* (Fig 6). This pattern is also evident in samples of *Cx*. *nigripalpus*, suggesting that ASVs *Lactococcus*, and *Aeromonas* are the ones that fluctuate significantly (Fig 6). In contrast, when *Wolbachia* levels exceed 30% in *Ev*. *dubitans*, *Mi*. *micropyga*, *Br*. *hamata*, and *Cx*. *nigripalpus*, and *Rickettsia* exceed 50% of the composition in *Pi*. *pia*, significantly reduces the relative abundance of ASVs listed above (e.g., *Ralstonia*, *Acinetobacter*, *Staphylococcus*, *Aeromonas*) (Fig 6).

The alpha diversity analysis is consistent with composition profiles, and we also observed a possible effect due mainly to host and abundance of *Wolbachia* (Fig 7A–7E) on the diversity of bacterial communities within sand flies and *Cx*. *nigripalpus*. The alpha diversity per host shows a value lower of Chao 1 and Observed for sand flies in the Carribbean ecoregion (Fig 7A and 7B). Samples as *Pi*. *pia* has these indeces were higher than the rest of phlebotomines or mosquitoes. *Cx*. *nigripalpus*, *Br*. *hamata* and *Pi*. *evansi* have the Shannon and Simpson indexes higher with respect to other sand flies (Fig 7C and 7D).

We found that *Wolbachia* infection decreased the diversity and number of taxa (*Observed = 50–200*), the adjusted richness (*Chao 1 = 50–200*) as well as the uniformity and/or distribution of ASVs in different samples, as seen in the *Shannon* index (Fig 7E). This impact of *Wolbachia* is associated with the Simpson index, which shows an increase in the dominance of ASVs, mainly in sand flies (Simpson index = 07–09). In contrast, the sand flies and *Cx*. *nigripalpus* that have other endosymbionts (*Cardinium*/*Microsporidia*) show a high diversity (*Shannon* index = 2–4.5; *Chao 1* index = 200–450), suggesting the possibility of finding more rare species in their niches and a high richness (Fig 7E).

The hierarchical analysis of clusters (Beta Diversity) derived from the comparison of ASVs at level of genus (Fig 8A), shows three groups (a = Sand flies and *Cx*. *nigripalpus* from Andean ecoregion; b = Sand flies from Caribbean region; c = Sand flies and *Cx*. *nigripalpus* from both ecoregions), suggesting that the composition of the prokaryotic microbiota is also determined for geographical distribution (ecoregion Andean or Caribbean), but also show in the influence of the host. The Principal Coordinate Analysis (PCoA) is consistent, which showed statistically significant differences (PERMANOVA, F = 2.4744; R2 = 0.18363; p-value = 0.007) between the microbiota of sand flies and mosquitoes depending on its origin and possibly for the abundance or presence of some endosymbionts (*Wolbachia*, *Rickettsia*) (Fig 8B). However, the NMDS plot was performed to assess significance among *Wolbachia* and bacterial community composition (Fig 8C), did not exhibit significant clustering in either between groups of sand flies or mosquitoes (PERMANOVA, F = 1.1429; R2 = 0.094118; p-value = 0.324; [NMDS] Stress = 0.11348). Only some samples, as *Cx*. *nigripalpus* and *Mi*. *Micropyga* showed differences visually significant on *Wolbachia* in this analysis. Now, the Kruskal–Wallis test, which showed statistically significant interspecific differences between the sand flies and mosquito total microbiota (***p<0.0005), using weighted and unweighted Unifrac distances.

**Fig 9:**
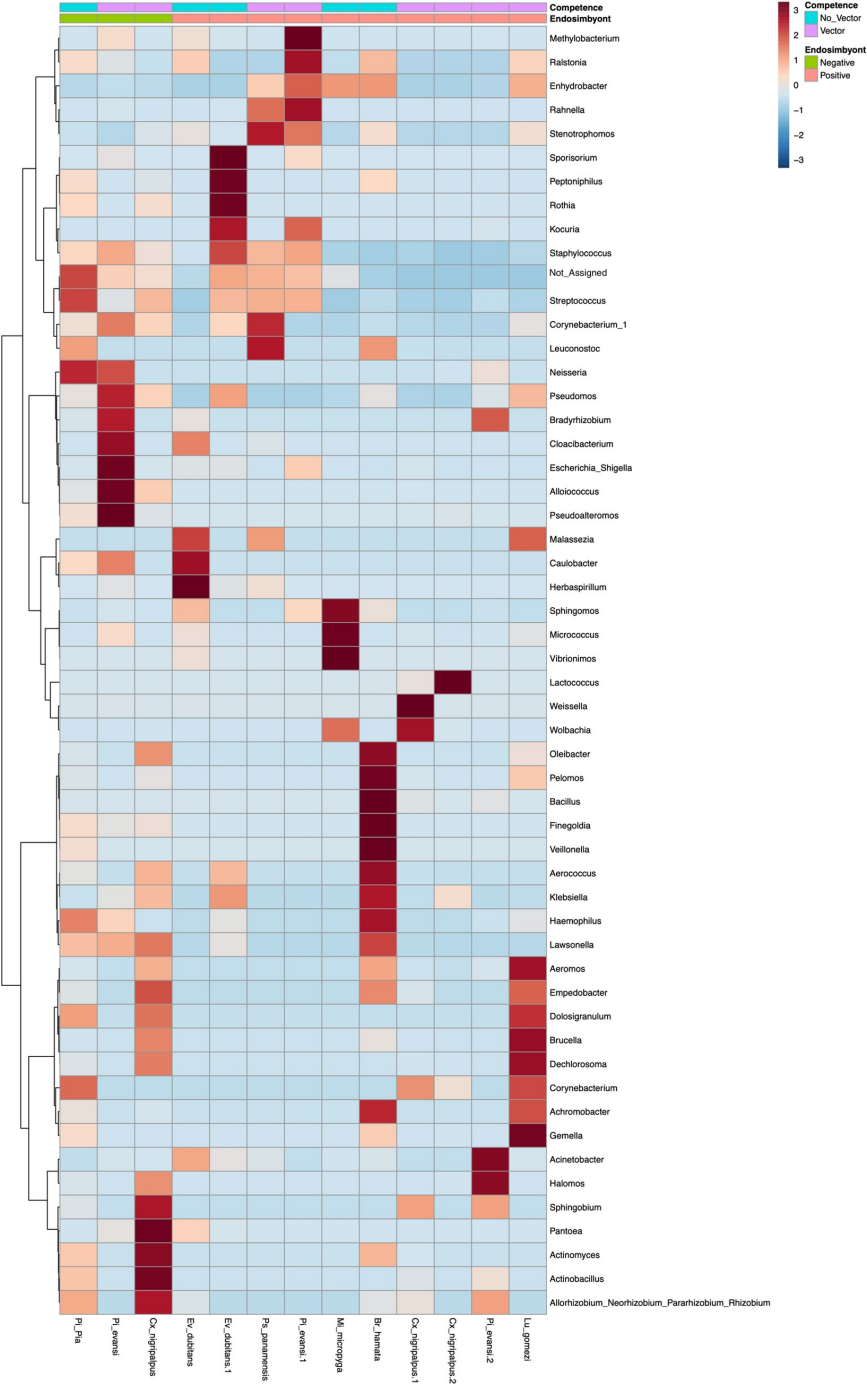
Heatmap based on the microbiota composition at the genus level illustrating common and endemic bacterial community abundances according to insects, the presence of endosymbionts and categorization according to its importance as a vector. Hierarchical Ward’s linkage clustering based on Pearson’s correlation coefficient of the microbial taxa abundance. Red and blue colors represent positive and negative correlations, respectively. The color scale represents the scaled abundance of each variable, denoted as Z-score, red indicates high abundance, and blue indicating low abundance.

The heatmap cluster analysis (Fig 9) at the genus level with 10 samples of sand flies and three samples of *Cx*. *nigripalpus* includes 54 bacterial genera were overlapped differentially according to all species (host). Between five and ten bacterial genera contributed 90% abundance of bacterial communities per sample of insects. No bacterial genus served as biomarkers were found in unique bacteria of sand flies or mosquitoes.

**Fig 10:**
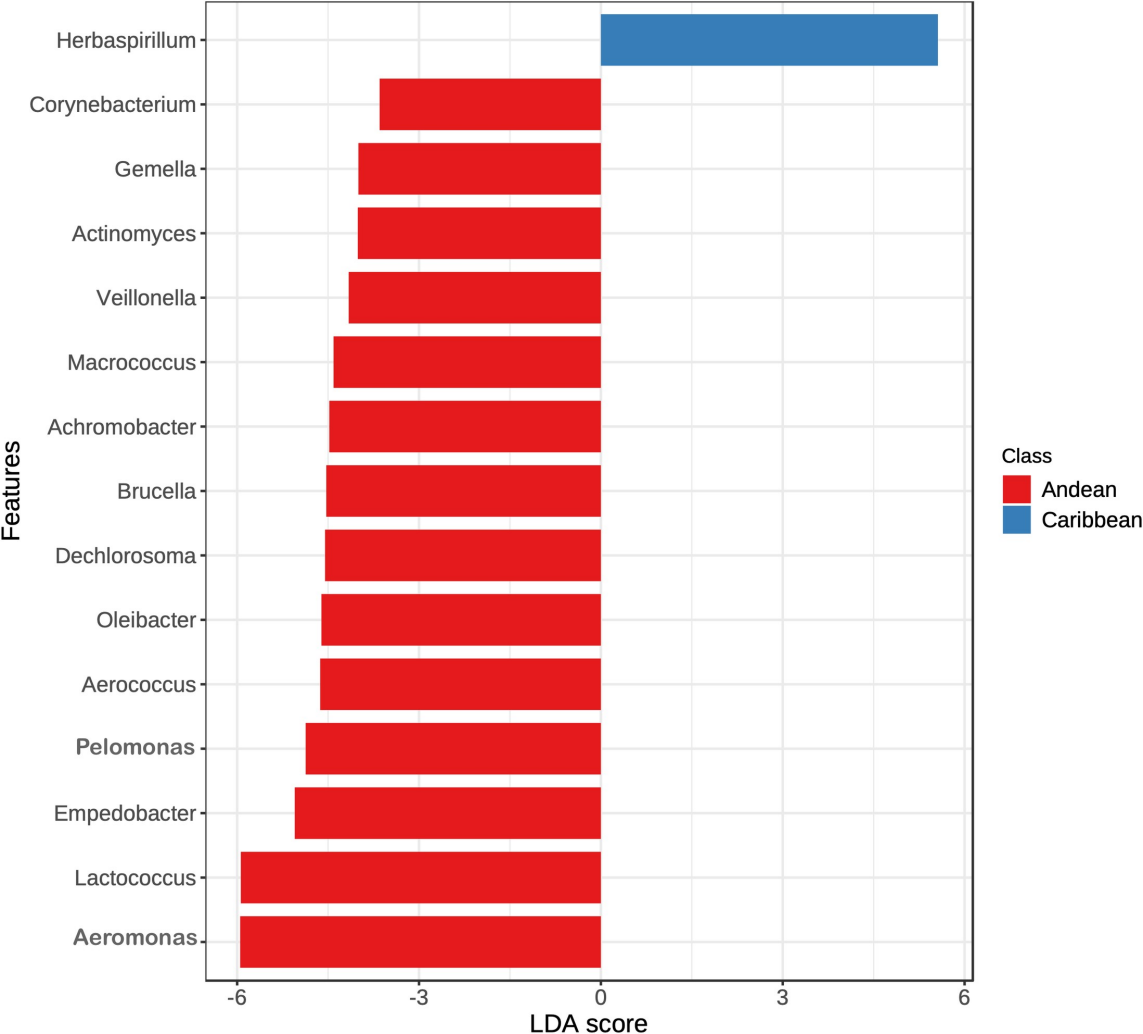
Linear discriminant analysis effect size (LEfSe) of gut microbiota in sand flies and *Culex nigripalpus* de Colombia.

The core microbiota of sand flies and *Cx*. *nigripalpus*, mainly show some ASVs (*Aeromonas*, *Ralstonia*, *Corynebacterium*, *Acinetobacter*, *Pseudomonas*) with relative abundance and prevalence significant (S5 Fig), and other taxa as *Lactococcus*, *Empedobacter* that has a consistent behavior with Linear discriminant analysis effect size (LEfSe), that was performed to identify specialized bacterial groups in sand flies and mosquitoes at each ecoregion. At genus level, the LEfSe showed 14 bacterial taxa with significant differences in insects of Andean ecoregion (Fig 10), such as *Aeromonas*, *Lactococcus*, *Empedobacter*, *Veillonella*, among others. Whereas *Herbaspirillum* was the ASVs most abundant and have significant differences in insects from Caribbean ecoregion (Fig 10).

**Fig 11:**
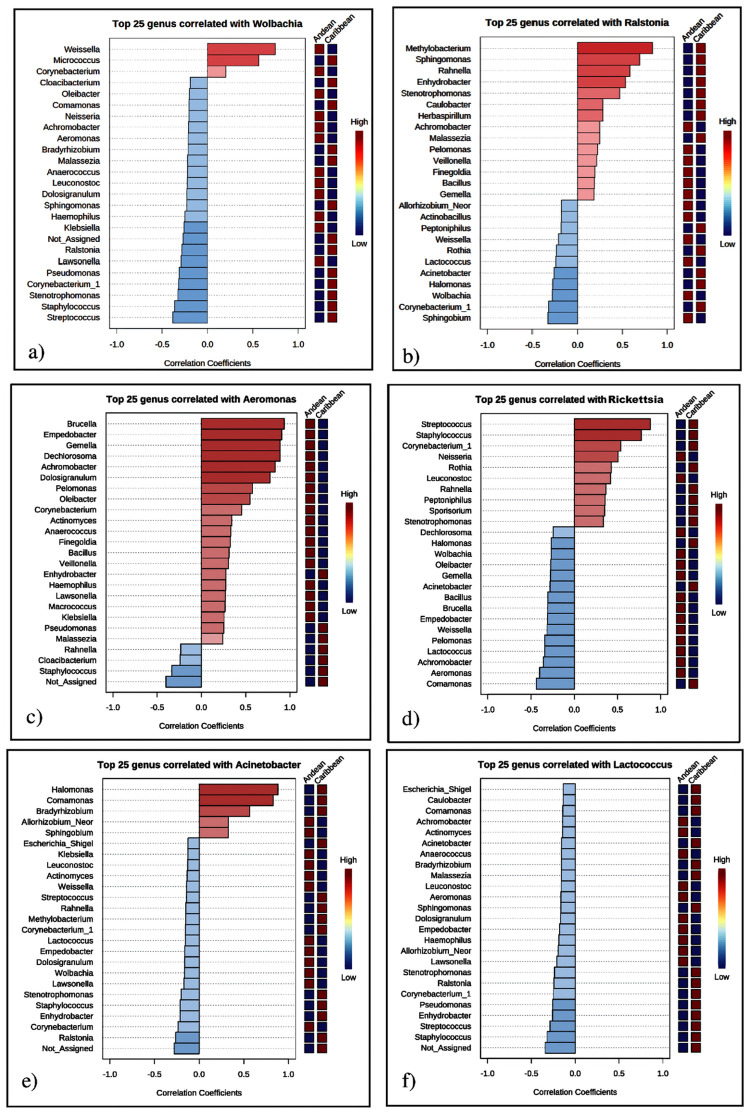
The random forest analysis shows positive interactions and significant features among some taxa in the core microbiota in sand flies and *Culex nigripalpus* de Colombia. The top 25 of ASVs most abundant are considered. The y axis, from top to bottom, displays the taxa ranked by their importance or association with other ASV and ecoregion.

The random forest analysis was highly reproducible and shows interactions among some taxa (*Wolbachia*, *Ralstonia*, *Rickettsia*, *Acinetobacter*, *Aeromonas*) that act as significant features in the microbiota in sand flies and *Culex nigripalpus* de Colombia (Fig 11). Accuracy and robustness of the random forest revealed that *Wolbachia* have positive interactions only with *Weisella*, *Micrococcus* and *Corynebacterium* (Fig 11A). ASVs of *Ralstonia* and *Aeromonas*, are also important in the structure and composition of the microbiota, for having a greater number of positive interactions, mainly with *Methylobacterium*, *Sphingomonas*, *Rahenella* for the case of *Ralstonia* and *Brucella*, *Empedobacter*, *Gemmella*, *Dechlorosoma Achromobacter* and *Dolosigranulim* for the case of *Aeromonas* (Fig 11B and 11C). The endosymbiont *Rickettsia* presented strong interaction with *Streptococcus* and *Staphylococcus* (Fig 11D), while that *Acinetobacter* presented strong interaction with *Halomonas* and *Comamonas* (Fig 11E). ASV of *Lactococcus* was abundant but show no interaction with other ASV the microbiota (Fig 11F).

A deep and specific search shows an ASV corresponding to *Candidatus cardinium*, only in *Pi*. *evansi* with low relative abundance (0.2%; Reads = 149) (Table 5) and show a higher pattern of relative abundance of the ASV *Microsporidia* in *Pi*. *pia* (23%; Reads = 136062) (Table 5), *Cx*. *nigripalpus* (11%; Reads = 38938) and *Pi*. *evansi* (0.2%; Reads = 1017). Also, the *Microsporidia* infection is revealed in *Ev*. *dubitans* (2%; Reads = 12663) and *Br*. *hamata* (0.1%; Reads = 240) (Table 5). Microsporidia was detected only at the level of Phylum (S4 Fig). Other endosymbionts, as such *Flavobacterium*, were found in *Pi*. *pia* (0.03%; Reads = 43), *Ev*. *dubitans* (0.34%; Reads = 2356) and *ps*. *panamensis* (0.8%; Reads = 3531) (Table 5). In *Cx*. *nigripalpus* (0.29%; Reads = 1386) and *Pi*. *pia* (0.03%; Reads = 160) ASVs of *Asaia*, were also determined (Table 5). We also showed the first *Bartonella* record in *Cx*. *nigripalpus* (Department of Antioquia) with a lower relative abundance (0.2%).

**Table 5:** Presence and number of reads (relative abundance) of several endosymbionts in the microbiota of sand flies and *Culex nigripalpus*. ASVs at the level of the genus.

	Number of reads for Endosymbiont						
Sample	*Wolbachia*	*Cardinium*	*Microsporidia*	*Rickettsia*	*Flavobacterium*	*Asaia*	*Arsenophonus*
*Cx*. *nigripalpus*	466591	12	38938	0	0	1386	205
*Lu*. *gomezi*	113447	0	0	0	0	0	0
*Br*. *hamata*	205915	0	240	0	0	0	0
*Pi*. *pia*	0	0	136062	268264	43	160	0
*Ev*. *dubitans*	815832	0	12663	0	2356	0	0
*Ps*. *panamensis*	0	0	0	0	3531	0	0
*Mi*. *micropyga*	143397	0	0	0	0	0	0
*Pi*. *evansi*	149	565	1017	0	0	0	0

We also explored the presence of archaea in sand flies and mosquito’s species (S6 Fig). Archaeal ASVs showed a low overall abundance relative to ASVs compared to bacteria. The most abundant ASVs belonged to Crenarchaeota (83%), Euryachaeota (16%), and Thaumarchaeota (1%) (S6 Fig). Crenarchaeota (Family Thermoproteaceae) was mainly found in *Ev*. *dubitans* (reads of ASV = 195) with 0.04% of total microbiota, while Euryachaeota (genus Halalkalicoccus and Halococcus) was found in *Pi*. *evansi* (ASV = 42; 0.01%). Crenarchaeota and Thaumarchaeota (Family Nitrososphaeraceae) also have a presence in *Pi*. *pia* but in low abundance.

## Discussion

Molecular approaches (direct PCR and 16S rRNA gene survey) and phylogenetic relationships of different secondary endosymbionts (*Wolbachia*, *Cardinium*, *Microsporidia*, *Rickettsia*, *Flavobacterium*) were estimated for natural populations of sand flies and mosquitoes in two ecoregions from Colombia, representing this research a current and focal overview of the association of endosymbionts and their hosts.

*Wolbachia* is the endosymbiont of a major impact at present in biological control principally of arbovirus [96], although recently its utility has been evaluated to impact the system immune of sand flies and *Leishmania* [12, 13]. In this study, sequences (*wsp* gene) of *Wolbachia* obtained from *Ev*. *dubitans* and *Mi*. *micropyga* of the Caribbean ecoregion (Cordoba department) showed high similarity with sequences of strains of the Leva group (*wLeva-wLcy*) of *Wolbachia* supergroup B, previously described in a local population of *Pi*. *evansi* from the department of Sucre [20, 23], a species that was also infected in this study but in another locality (Sabanas del Potrero). We report the first *Wolbachia* record in *Mi*. *micropyga* and the high frequency of infection in *Ev*. *dubitans*. It can be established that strains of the *Wolbachia* “Leva group” are closely associated and distributed among the sand flies of the Caribbean region. Interestingly, some strains of Supergroup B (*wPip*) have phenotypes associated with cytoplasmic incompatibility, resulting commonly in between-population infertile mating [97]. Considering the frequency, distribution, and potential phenotype of *Wolbachia* in the Caribbean region, we suggest that they might be used for establishing biological control strategies that impact leishmaniasis transmission by sand flies.

In the Andean region (Antioquia department) the natural infection of *Wolbachia* in sand flies is recorded for the first time and reports on two new strains in *Lu*. *gomezi* and *Br*. *hamata* called *wGom* and *wBham*, respectively. These two strains of *Wolbachia* were located in supergroup A, close to wOri and wCauB strains. *Lu*. *gomezi* has an epidemiological impact as a vector of cutaneous leishmaniasis in different regions of Colombia [98]. About *Br*. *hamata*, a non-vectoring species of leishmaniasis, the finding is interesting because the range of *Wolbachia* is extended in species of the subfamily Phlebotominae. In Colombia, the frequency and abundance of *Brumptomyia* collection are low and associated with localities of vegetated forest preserved [99].

Sampling and analysis of mosquitoes in the department of Antioquia allowed also to confirm the natural infection of *Wolbachia* in adult females of *Cx*. *nigripalpus* and establish a new group called Cnig (supergroup A) by analyzing the *wsp* gene. *Cx*. *nigripalpus* had been reported with *Wolbachia* in a population of Florida (USA) using a 16S rRNA gene survey, which only provides resolution until the genus level [100]. *Cx*. *nigripalpus* is a primary vector of the Saint Louis Encephalitis (SLE) virus in North America and an important vector for other arboviruses, such as West Nile virus (WNV) in the southeastern United States [101]. *Wolbachia* in mosquitoes of the genus *Culex* (WNV vectors) varies according to the seasons and infections are negatively correlated with WNV in these mosquitoes [102].

*Cx*. *nigripalpus* was also naturally infected with *Microsporidia*, as was *Pi*. *pia* and *Pi*. *evansi*. Different species of *Microsporidia* (*Amblyospora* and *Vavraia*) are strongly associated with mosquitoes (*Aedes*, *Ochlerotatus*, *Culex*, *Psorophora*, *Wyeomyia*, *Anopheles*) on the continental level [103, 104], but with few findings in sand flies [64]. Previously, *Cx*. *nigripalpus* from Florida (USA) had been reported with *Amblyospora* sp. 1, which is located in the *Culex* group within the *Aquasporidia* class [103]. In contrast, our study indicates that *Microsporidia* of *Cx*. *nigripalpus* (Andean Ecoregion) is phylogenetically located in the Terresporidia class, with the sequences reported for the first time for the natural populations of *Pi*. *pia*, suggesting a possible association by geographic distribution and not by the type of environment. Molecular analysis suggests that *Microsporidia* of *Pi*. *pia* and *Cx*. *nigripalpus* corresponds to a news strain closely related to *Microsporidia* MB, which is vertically transmitted and blocks the transmission of *Plasmodium falciparum* in *An*. *arabiensis* [59]. This suggests that it could be further explored as a new parasite control tool, including *Leishmania*.

This study also provides new knowledge on natural *Cardinium* infection and demonstrates the phylogenetic location of different strains in groups C and A, obtained from the DNA of *Tr*. *triramula*, *Ps*. *shannoni*, and *Mi*. *cayennensis*, respectively. Previously *Pi*. *evansi* in the Caribbean region was registered with *Cardinium* [23]. In this research was detected an individual with double infection (*Wolbachia* and *Cardinium*). The ecological and metabolic effects of coinfections are not well understood, although a model of possible *Wolbachia–Cardinium* interaction is proposed with possible complementation in function for pathways such as methionine and fatty acid biosynthesis and biotin transport in Nematode [105].

The sequences are categorized as the *Cardinium* of *Tr*. *triramula* and *Ps*. *shannoni* located in group C, indicating a close relationship with *Cardinium* of different *Culicoides* species, but they are located in group A of *Pi*. *cayennenis* and *Pi*. *evansi* form two genetic variants and distant from the strains reported within this group. The global diversity of this reproductive parasite in their natural contexts and the interactions with their hosts, thus, remains to be characterized in depth [37] and demand further study that includes incidence, prevalence, and geographical variation between natural populations of Phlebotominae sand flies. However, the bacterial beneficial abilities of insect hosts are probably related to *Cardinium* motility and toxin synthesis [106]. Due to the motility feature, *Cardinium* may directly contact a host’s parasitoid or pathogens and kill it by secreting toxins [106]. Also, the *Cardinium*-free insects had higher fitness than *Cardinium*-infected insects [107].

*Wolbachia* and *Microsporidia* have a higher frequency of natural infection in the Caribbean region, but their frequency decreases in the Andean region significantly, in contrast to the behavior of *Cardinium*. Several studies show that *Wolbachia* significantly and efficiently reduces the proportions of mosquitoes achieving infection and transmission potential of pathogens across the different regions [102]. The frequency of infection by *Wolbachia* also is sensitive to temperature environmental variations [108], and *Cardinium* infection showed a clear and consistent tendency to increase with temperature in terrestrial arthropods [14], suggesting that differences between species can exist [109]. Although our study shows preliminary and similar behavior, it may be better when considering a greater number of samples, taxonomic scales, the inclusion of other Colombian regions (Orinoquia, Amazonia, Pacifico), as well as the relationship with the natural infection by *Leishmania* or viruses in vector insects to have a global analysis.

Now, a sand flies population may have a high frequency of infection, but the low density of endosymbionts and this characteristic can have role important in the expression of the reproductive phenotype (as cytoplasmic incompatibility) or limits the degree of parental spread, impacting the design of biological control strategies. The infection levels of endosymbionts vary widely among populations of insects and are also influenced by the dynamics and interactions with the microbiota, justifying the inclusion of more sensitive techniques such as MiSeq Illumina for better understanding. These interactions have serious implications for the fitness, ecology, and evolution of insects [110].

*Pi*. *evansi* have a high number of infected individuals with *Wolbachia*, but a low density (0.04%) according to the number of ASVs obtained from this endosymbiont in the total microbiota, in contrast to *Lu*. *gomezi*, *Ev*. *dubitans*, *Mi*. *micropyga*, *Br*. *hamata*, and *Cx*. *nigripalpus*, which presented abundance relative between 30% and 83%. This variation in *Wolbachia* density directly can influence the composition, structure, and diversity of microbiota, and especially the fluctuation of other ASVs (*Acinetobacter*, *Lactococcus*), also indicating a clear influence of the host species on the composition of prokaryotic microbiota mainly in sand flies. Papadopoulos et al., 2020 in *Phlebotomus* sand flies (*P*. *papatasi*, *P*. *neglectus*, *P*. *tobbi*, *P*. *similis*), also indicates that the host genotype and presence of endosymbionts are the major modulators of gut microbiota [110].

Insect vectors also use the microbiome as a rapid evolution mechanism to adapt to rapidly changing environmental conditions [111]. In this context, *Flavobacterium* was found in *Ev*. *dubitans* and *Ps*. *panamensis*. This secondary endosymbiont has not yet been studied in sand flies of America and is of great relevance in various insect families because of its horizontal transfer and generating the sex ratio distortion [112], indicating that its presence can promote changes in the microbiota of the host and increase its capacity of adaptation for new environments [113]. Also, been recorded that there is a negative correlation when it co-exists with other endosymbionts (e.g., *Wolbachia* and *Spiroplasma*), moving from their hosts [113].

A detailed analysis of the microbiota allowed us to determine for the first time the natural infection of pathogens of medical importance, such as *Rickettsia* sp. in natural populations *Pi*. *pia* (Department of Caldas, Municipality of Salamina) with a relative abundance of this ASV of 58,8%. Reeves et al., 2008 reported to *Lutzomyia apache*, a North American sand fly of the subgenus *Helcocyrtomyia* with *Rickettsia sp*. using PCR conventional and was similar to the *Rickettsia* endosymbiont of a spider, the sister taxa to the spotted fever and typhus group *Rickettsia* [114]. Two wild populations from China of *Phlebotomus chinensis* were also reported with the endosymbionts *Rickettsia* [48]. This is the first report by *Rickettsia* for sand flies from Colombia, which are obligate endosymbionts identified mainly in ticks [115].

Few studies have been conducted for detecting *Bartonella* in arthropods in Colombia. *Cx*. *nigripalpus* result infected by *Bartonella* sp. The genus *Bartonella* is a group of bacteria that are selective mammalian pathogens requiring specific vectors to be transmitted (as sand flies) and causes Carrion’s disease in mountainous regions of South America, in countries as Ecuador and Peru [116–118]. *Bartonella* has been reported previously in medically important mosquitoes such as *Aedes vexans*, *Culex pipiens*, *Anopheles maculipennis* [118].

We evidence the presence of archaea ASVs in sand flies of América, as *Ev*. *dubitans*, *Pi*. *pia* and *Pi*. *evansi*. Was found low abundance relative (0.01%-0,04%) of archaea, concerning 3% reported in other insects as termite, *Spodoptera*, and Coleoptera [119], but similar to the values registered in *Phlebotomus* sand flies (<0.01%) [110]. Only the ASV Thaumarchaeota (Family Nitrososphaeraceae) was similar between populations of sand flies from Colombia and *Phlebotomus* [110]. The presence of archaea may suggest the establishment of anaerobic zones in the intestine of phlebotomines, and these communities may be acquired from the soil during the larva phase and have a decisive role in biochemical processes (e.g., oxidation of ammonium to nitrite, pH adaptation) necessary for survival [120].

This study has several limitations. A low frequency of endosymbiont infection was determined in the group of insects analyzed. This suggests the need to increase the number of individuals per species and expand the geographical representation of origin of sand flies and mainly mosquitoes. Also, standard PCR of *wsp* gene frequently produced false negatives and positives [121]. Some PCR products of *Wolbachia* found in mosquitoes or sand flies (e.g., *wsp* of *An*. *trianulatus; wsp* of *Pi*. *evansi* Fig 2C, Lane 11) were unclear, smaller bands in lanes in some samples were non-specifical PCR product, which was more evident during the analysis of the sequences. For this reason, some products of *wsp* gene were excluded from the study. These results may imply a difference in the density of *Wolbachia* infecting the hosts, the presence of two genetic variants (co-infection of strains) that can be found naturally in the same sample, or perhaps because the DNA from the arthropod host interfered with amplification by Taq DNA polymerase [122]. These findings indicate also that reliable PCR detection of *Wolbachia* requires the use of other additional primers as 16S rDNA and *fts*Z [123]. However, our study included complementary analysis to generate amplicons of the hypervariable region V4 of the 16S rRNA gene by Illumina MiSeq sequencing to confirm the presence and abundance of endosymbionts.

## Conclusions

One of the main motivating forces for the study of vector microbiota is the possibility of identifying microorganisms potentially useful for developing control strategies. We provide information from new records of natural infection of secondary endosymbionts, such as *Wolbachia*, *Cardinium*, *Microsporidia*, *Flavobacterium*, and *Rickettsia* in insects recognized for harboring or transmitting parasites or arbovirus. Specifically, this study determined the identity and phylogenetic location of some strains of these endosymbionts. Also, it was possible to determine whether *Wolbachia* abundance and insect species are important in the patterns of the microbiota. Findings of epidemiological interest are immersed in this research work by the detection of *Bartonella* sp. and *Rickettsia* sp.

Some biological control mechanisms derived from our findings suggest the need to isolate endosymbionts such as *Wolbachia* and *Microsporidia* mainly. To know the location of these endosymbionts in determining organs of these insect vectors and their interaction with pathogens (parasites and viruses) important in public health is also necessary. Because of the high rates of infection of these endosymbionts, more studies are necessary to know if they influence the immune system of their hosts. With the information obtained as a base line, it is possible to obtain colonies of sand flies (as *Pi*. *evansi*, *Lu*. *gomezi*) or mosquitoes (as *Culex nigripalpus* or *Aedes*) with these endosymbionts through backcrossed and evaluate using in-vivo trials the possibility of establishing reproductive phenotypes and/or their ability to influence the vector competence of these important vectors to block or interrupt the development of pathogens.

## Supporting information

S1 Table Summary of results obtained from 16S rRNA gene amplicon sequencing of microbiota associated with sand flies and *Cx*. *nigripalpus* collected from several locations from Colombia. (DOCX)

S2 Table ASV table, metadata and taxonomic classification of the microbiota obtained of sand flies and *Culex nigripalpus* found with endosymbiont in several regions or departments from Colombia. (XLSX)

S1 Fig Agarose gel electrophoresis of PCR products of different bacterial endosymbionts, amplified from the total DNA of sand flies and mosquitoes (*Culex* and *Anopheles*) samples from Colombia. (DOCX)

S2 Fig Heterogeneity and variability for an internal group of sequences that include endosymbionts from sand flies and mosquitoes. (DOCX)

S3 Fig Library Size and detailed graphical summary of read counts calculated for each sample (a) and rarefaction curve from Chao1 analysis using partial 16S rRNA gene sequences from sand flies and *Cx*. *nigripalpus* collected of several locations from Colombia. (DOCX)

S4 Fig Gut microbiota composition in wild specimens of several natural population of phlebotominae sand flies and *Cx*. *nigripalpus*. Relative abundance of ASVs that were called to the taxonomic rank of Phylum. (DOCX)

S5 Fig Core microbiota of phlebotominae sand flies and *Cx*. *nigripalpus*. (DOCX)

S6 Fig The relative abundance of the most dominant archaeal ASVs (Phylum Level) detected in the sand flies. (DOCX)
