# Supplementary material for: Molecular phylogeny of heritable symbionts and microbiota diversity analysis in phlebotominae sand flies and Culex nigripalpus from Colombia
Source: PLoS Negl Trop Dis. 2021 Dec 20;15(12):e0009942. doi: 10.1371/journal.pntd.0009942 (PMC8722730; doi:10.1371/journal.pntd.0009942)
Supplement: S1 Table — (DOCX) [file pntd.0009942.s001.docx]

**S1 Table**: Summary of results obtained from 16S rRNA gene amplicon sequencing of microbiota associated with sand flies and *Cx. nigripalpus* collected of several locations from Colombia. **NA.** ASVs without taxonomic assignment.

| Summary | Sand flies and Mosquitoes-  Dataset untreated | Sand flies and Mosquitoes-  Dataset treated |
| --- | --- | --- |
| Total reads | 5’845.629 | 5’630.252 |
| # ASVs | 2054 | 550 |
| Phyla | *(28)*  *Acidobacteria*  *Actinobacteria*  *Apicomplexa*  *Bacteroidetes*  *Basidiomycota*  *Chlamydiae*  *Chloroflexi*  *Choanoflagellida*  *Ciliophora*  *Cloacimonetes*  *Crenarchaeota*  *Cyanobacteria*  *Deinococcus – Thermus*  *Epsilonbacteraeota*  *Eugelnozoa*  *Euryarchaeota*  *Firmicutes*  *Fusobacteria*  *Gemmatimonadetes*  *Hydrogenedentes*  *Microsporidia*  *Parabasalia*  *Patescibacteria*  *Protosteliida*  *Retaria*  *Spirochaetes*  *Thaumarchaeota*  *Verrucomicrobia* | *(17)*  *Acidobacteria*  *Actinobacteria*  *Bacteroidetes*  *Chlamydiae*  *Chloroflexi*  *Ciliophora*  *Cyanobacteria*  *Epsilonbacteraeota*  *Firmicutes*  *Fusobacteria*  *Microsporidia*  *Patescibacteria*  *Planctomycetes*  *Proteobacteria*  *Protosteliida*  *Spirochaetes*  *Verrucomicrobia* |
| Top 5 Family (total counts) | *Anaplasmataceae*  *Streptococcaceae*  *Moraxellaceae*  *NA*  *Rickettsiaceae* | *Anaplasmataceae*  *Streptococcaceae*  *Moraxellaceae*  *NA*  *Rickettsiaceae* |
| Top 5 genera (total counts) | - *Wolbachia* - *Lactococcus* - *Acinetobacter* - *NA* - *Rickettsia* | - *Wolbachia* - *Lactococcus* - *Acinetobacter* - *NA* - *Rickettsia* |
| # of taxa summarized to the genus level | 406 | 189 |
