# Supplementary material for: Molecular phylogeny of heritable symbionts and microbiota diversity analysis in phlebotominae sand flies and Culex nigripalpus from Colombia
Source: PLoS Negl Trop Dis. 2021 Dec 20;15(12):e0009942. doi: 10.1371/journal.pntd.0009942 (PMC8722730; doi:10.1371/journal.pntd.0009942)
Supplement: S1 Fig — (DOCX) [file pntd.0009942.s003.docx]

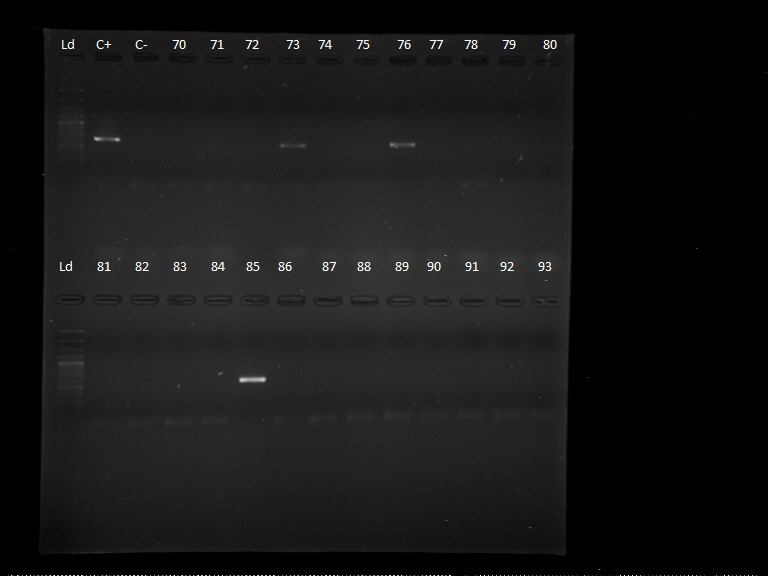

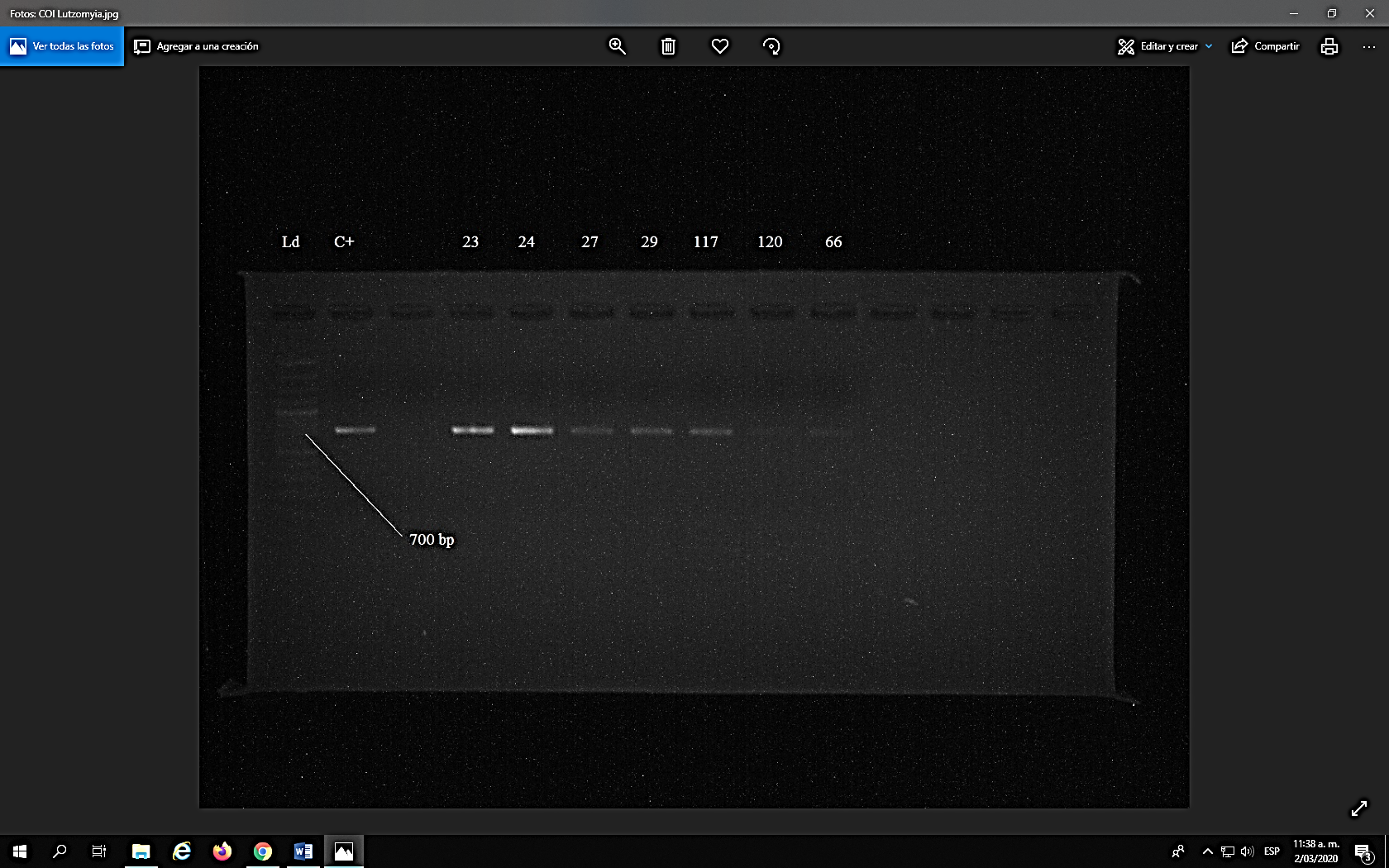


*****

*****

*****

a)

b)

Ld C+ C- 23 24 25 29 117 120 66

*****

650 bp


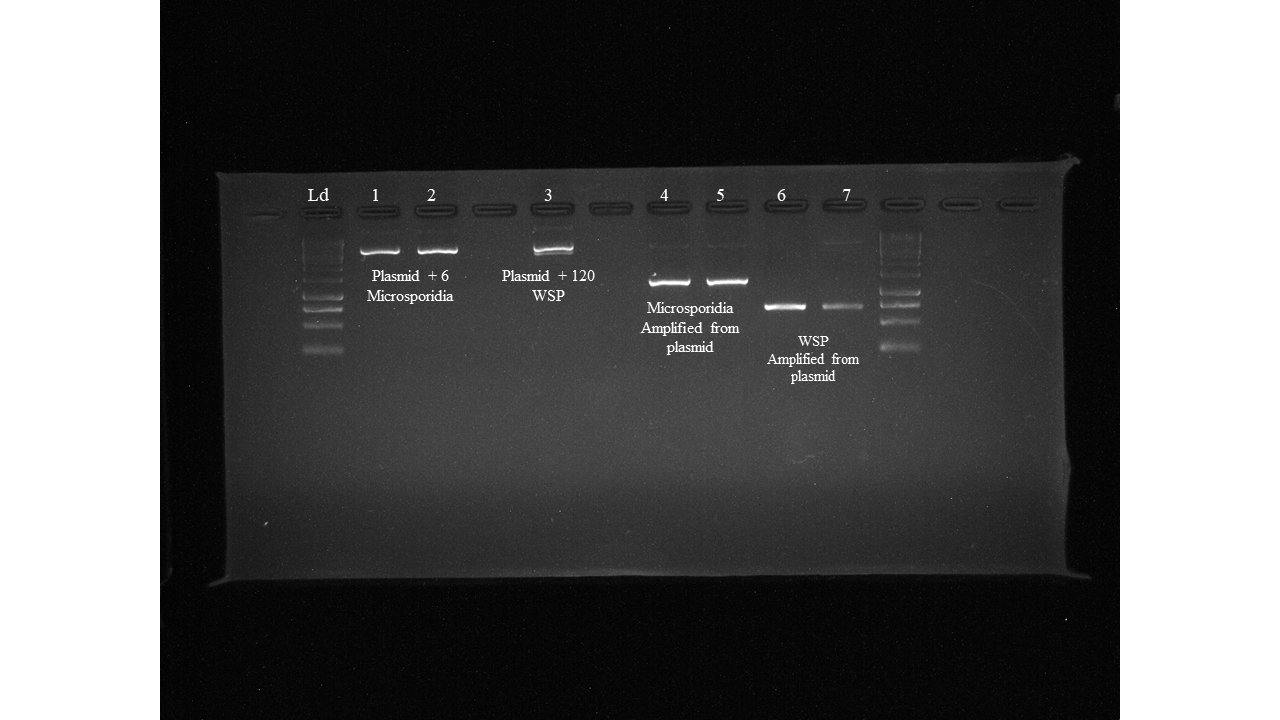

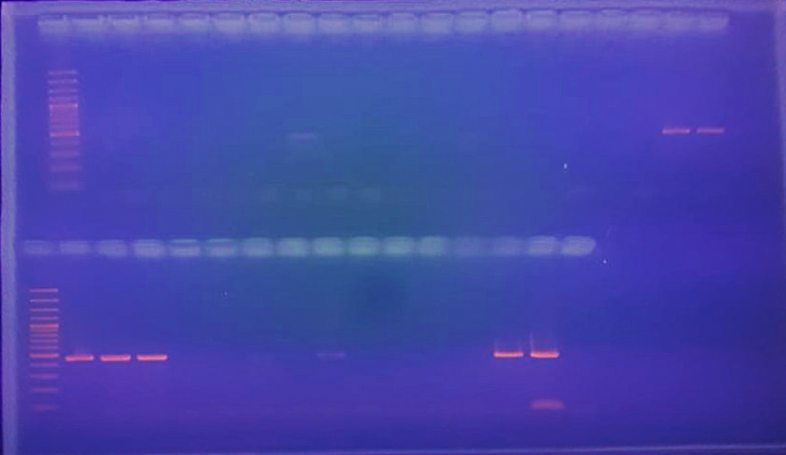


*****

*****

g)

h)

440 bp

Ld 136 137 138 139 140 141 14 150 151 152 153 154 C+ C+ C-


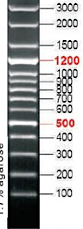


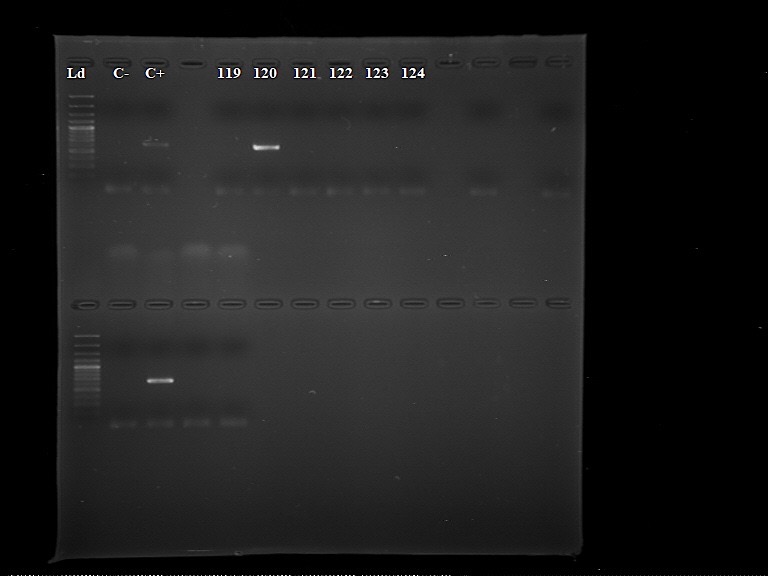

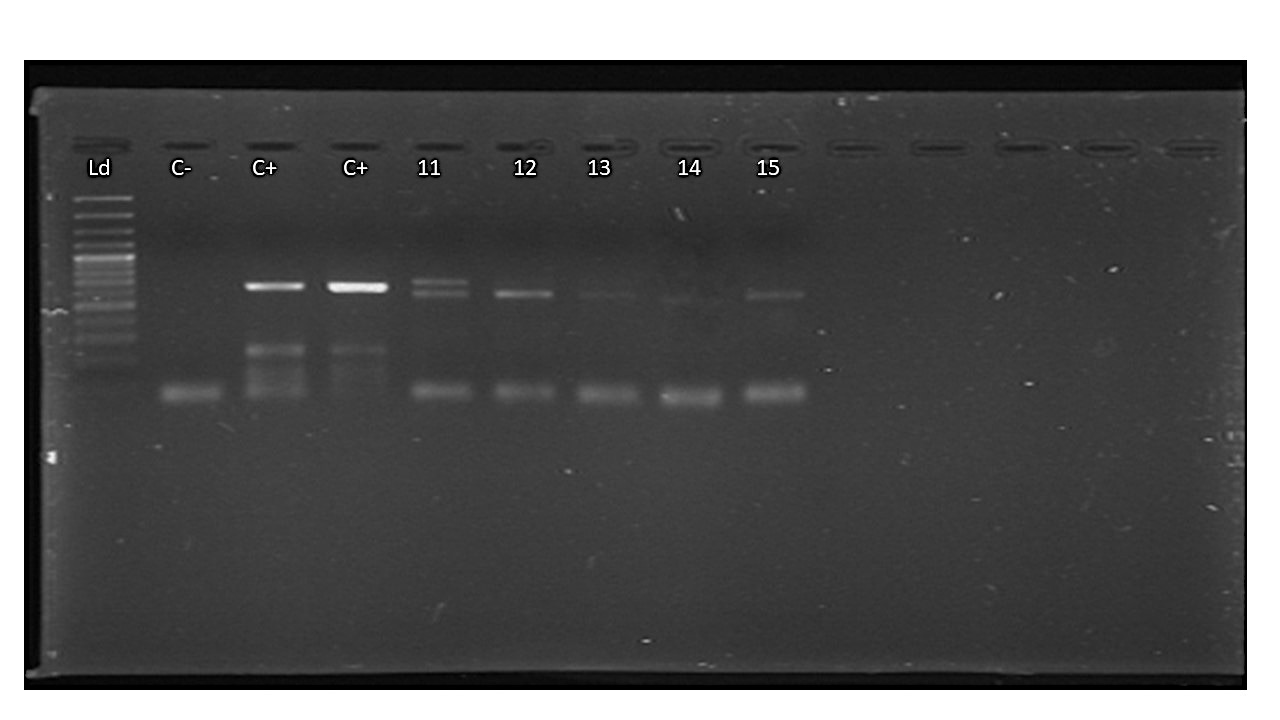


*****

*****

*****

*****

c)

d)

650 bp

650 bp


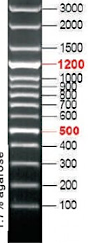


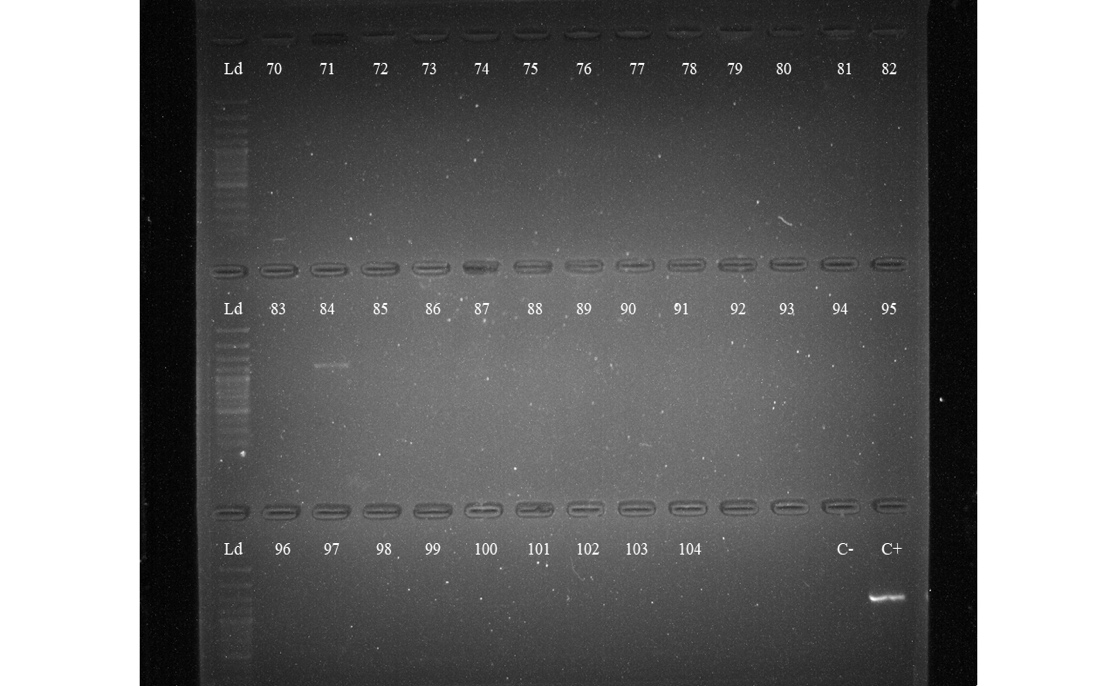


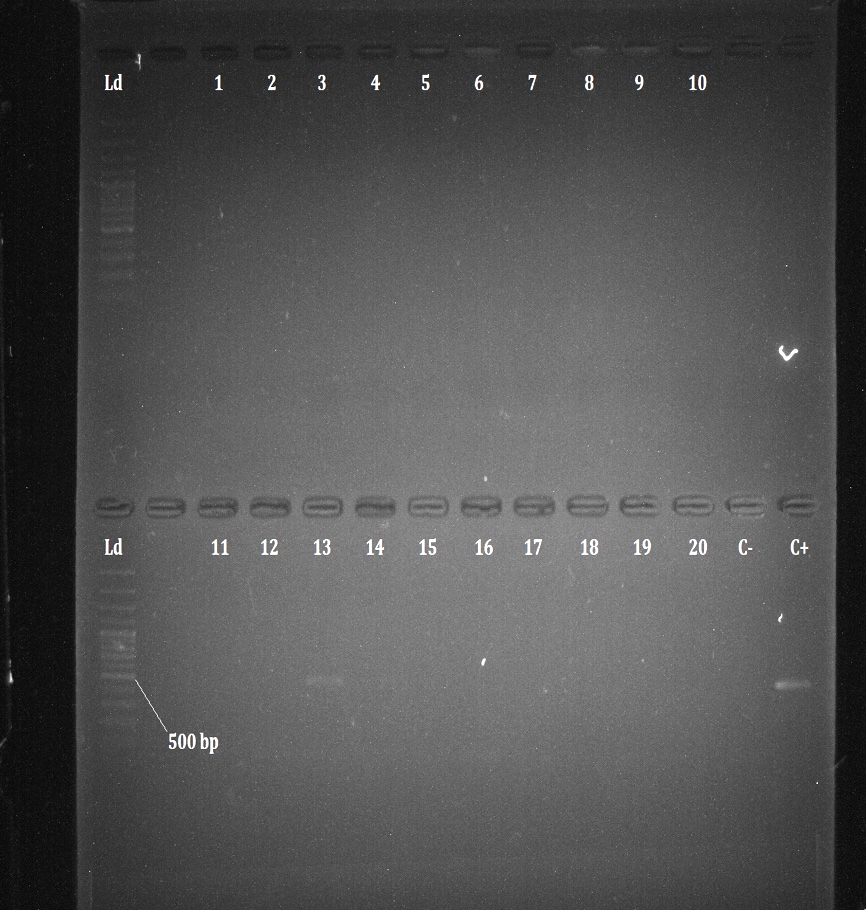


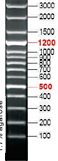


*****

*****

1500 bp

f)

440 bp


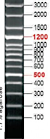


e)


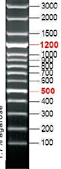


**S1 Fig.** Agarose gel electrophoresis of PCR product of different bacterial endosymbionts, amplified from total DNA of sand fly and mosquitoes (*Culex* and *Anopheles*) samples from Colombia. a) Represents PCR results for region of the COI (700 bp fragment) of positives samples for endosymbionts and to evaluate the quality of DNA and absence of inhibitors from genomic DNA. b) Represents PCR results for *Wolbachia* (650 bp) in *Culex nigripalpus*, *Pintomyia evansi* (c) and *Lutzomyia gomezi* (d). e) Represents PCR results for Microsporidia (1500 bp) in *Culex nigripalpus*. f) Represents PCR results for *Cardinium* (440 bp) in *Pintomyia evansi*, and *Micropygomyia cayennensis*. h) Represents the cloning of DNA fragment for *Wolbachia wsp* gene and the small subunit ribosomal DNA of Microsporidia obtained of samples from sand flies. *Spiroplasma* not was detected from any of the samples from any location. Abbreviations: Ld, a 100 bp DNA ladder; C-, negative control; C+, Positive control; White asterisk indicates positive PCR samples. yellow asteriskindicates a sample of *Pintomyia evansi* doubly infected with *Wolbachia* and *Cardinium.*
