# Supplementary material for: Molecular phylogeny of heritable symbionts and microbiota diversity analysis in phlebotominae sand flies and Culex nigripalpus from Colombia
Source: PLoS Negl Trop Dis. 2021 Dec 20;15(12):e0009942. doi: 10.1371/journal.pntd.0009942 (PMC8722730; doi:10.1371/journal.pntd.0009942)
Supplement: S2 Fig — (DOCX) [file pntd.0009942.s004.docx]

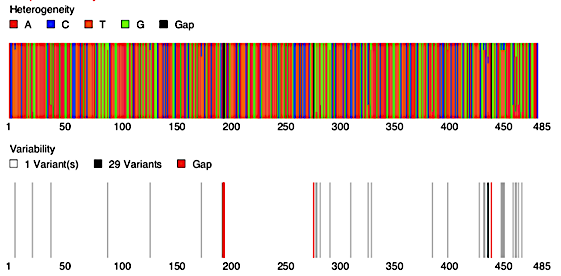

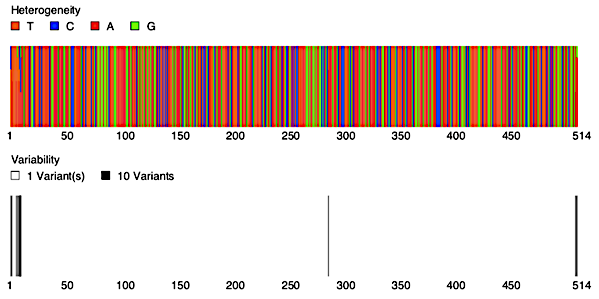


b)

a)

**
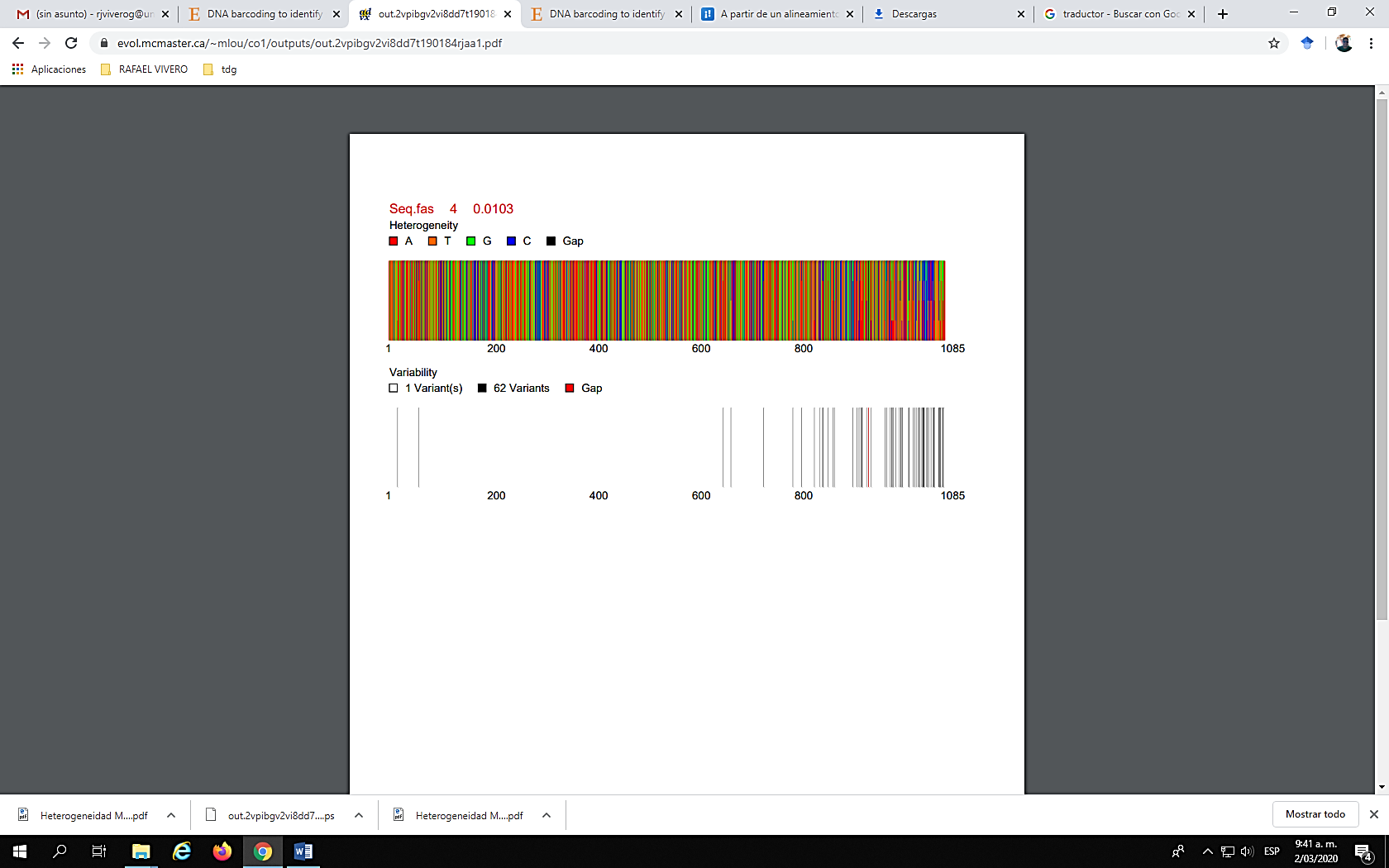
**
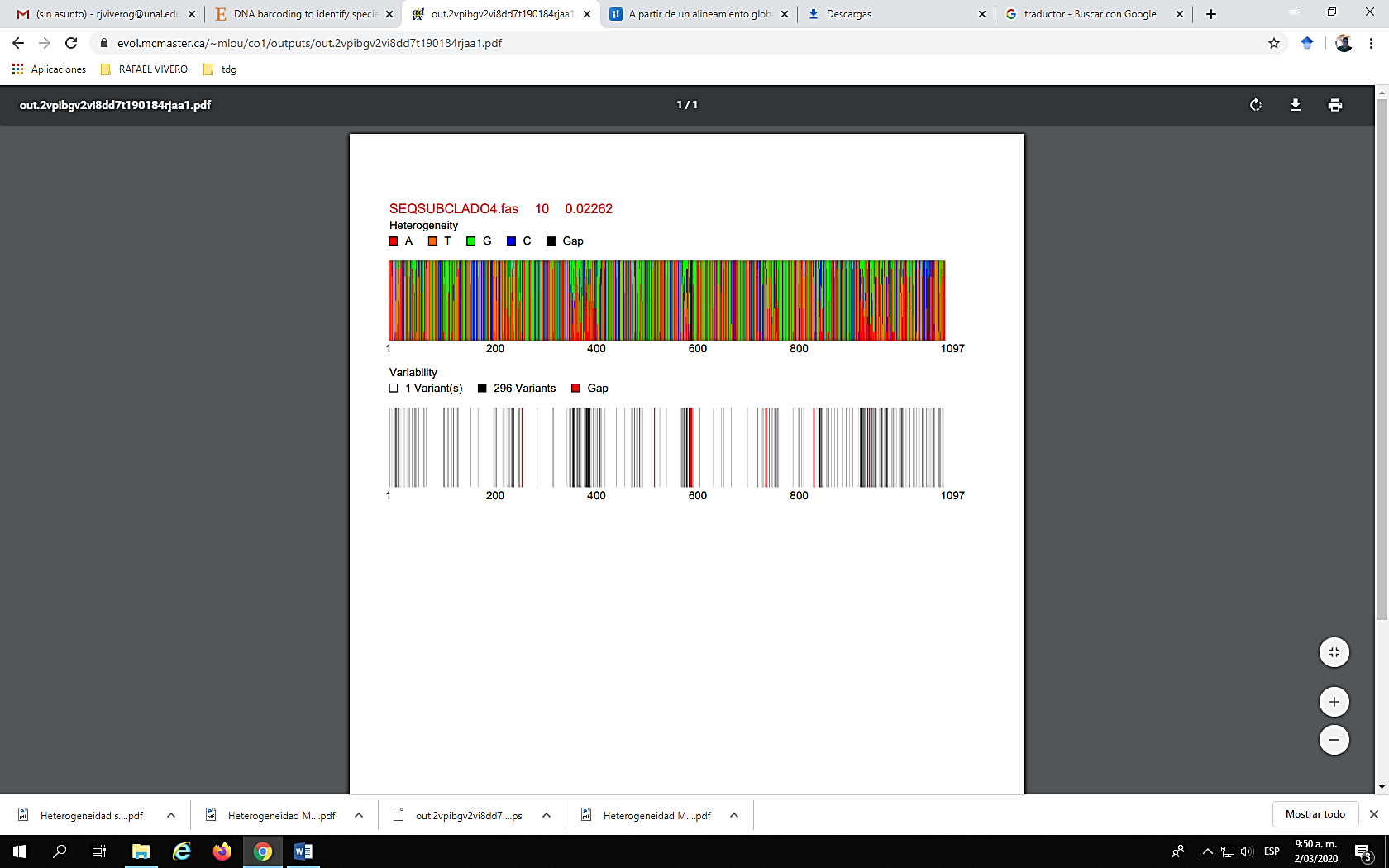


d)

c)


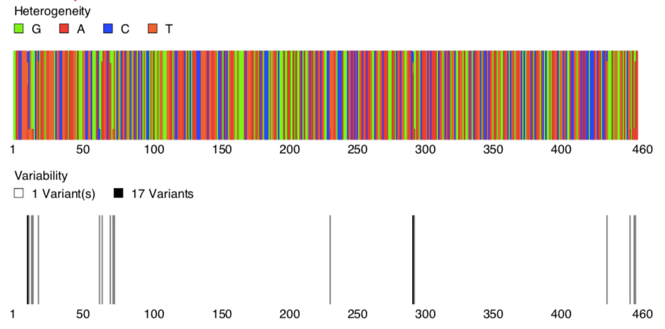

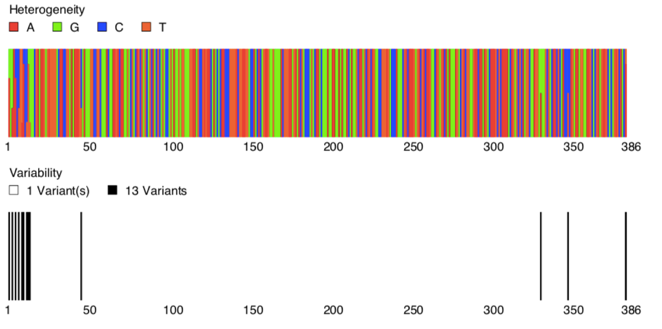


f)

e)


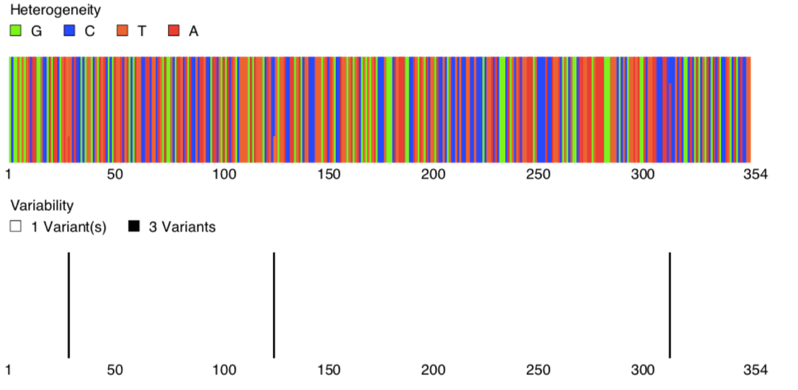


g)

**S2 Fig.**  Heterogeneity and variability for an internal group of sequences that include endosymbiont’s from sand flies and mosquitoes. a) Group *wCnig* of *Wolbachia* (sequences of *wsp* gene) only with members from *Cx. nigripalpus,* b) Group *wLeva* of *Wolbachia* (sequences of *wsp* gene) only with members of *Ev. dubitans* and *Mi. micropyga,* c) *Microsporidia* from *Culex*, *Lutzomyia pia* and *Microsporidian MB* and other microsporidia of Clade IV (sequences of the SSU rRNA), d) Microsporidia only from *Culex*, *Lutzomyia pia* and *Microsporidian MB*, e) *Cardinium* endosymbiont’s (sequences of 16S rDNA) only with members of group C that include Sand flies and *Culicoides* host´s, f) *Cardinium* endosymbiont’s only with members of subgroup 1 from *Mi. cayennensis*, g) *Cardinium* endosymbiont’s only with members of subgroup 2 from *Mi. cayennensis* and *Pi. evansi*.
