# Supplementary material for: Molecular phylogeny of heritable symbionts and microbiota diversity analysis in phlebotominae sand flies and Culex nigripalpus from Colombia
Source: PLoS Negl Trop Dis. 2021 Dec 20;15(12):e0009942. doi: 10.1371/journal.pntd.0009942 (PMC8722730; doi:10.1371/journal.pntd.0009942)

**S3 Fig.** Library Size and detailed graphical summary of read counts calculated for each sample (a) and rarefaction curve from Chao1 analysis using partial 16S rRNA gene sequences of sand flies and *Cx. nigripalpus* collected of several locations from Colombia. Saturated rarefaction curve indicates that the vastness of microbial diversity was retrieved from each sample.

**
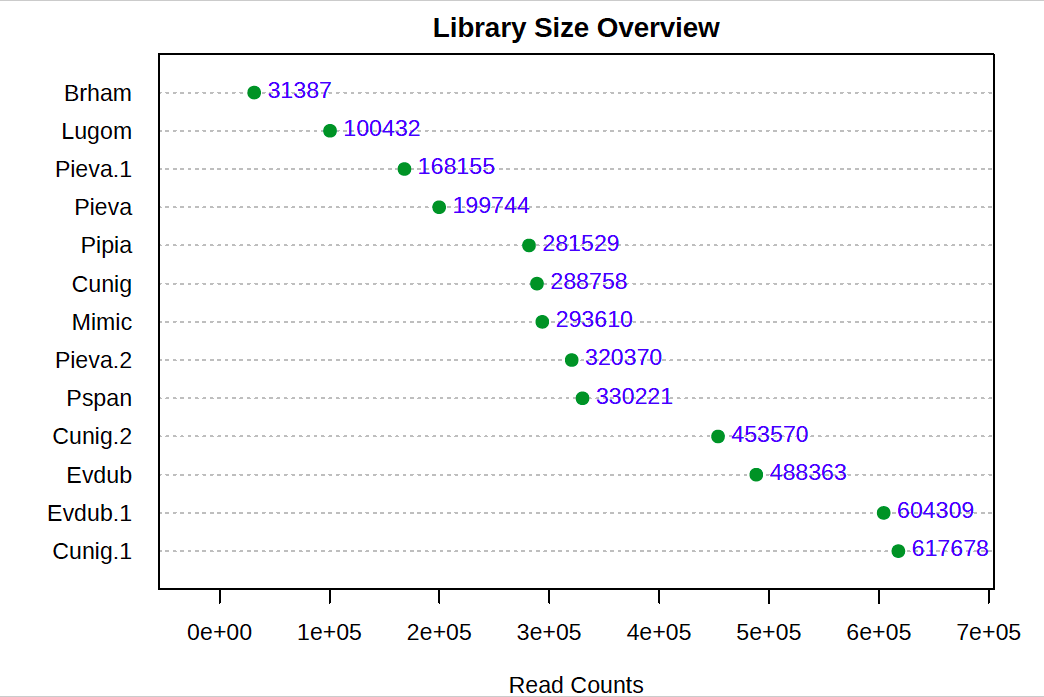
**


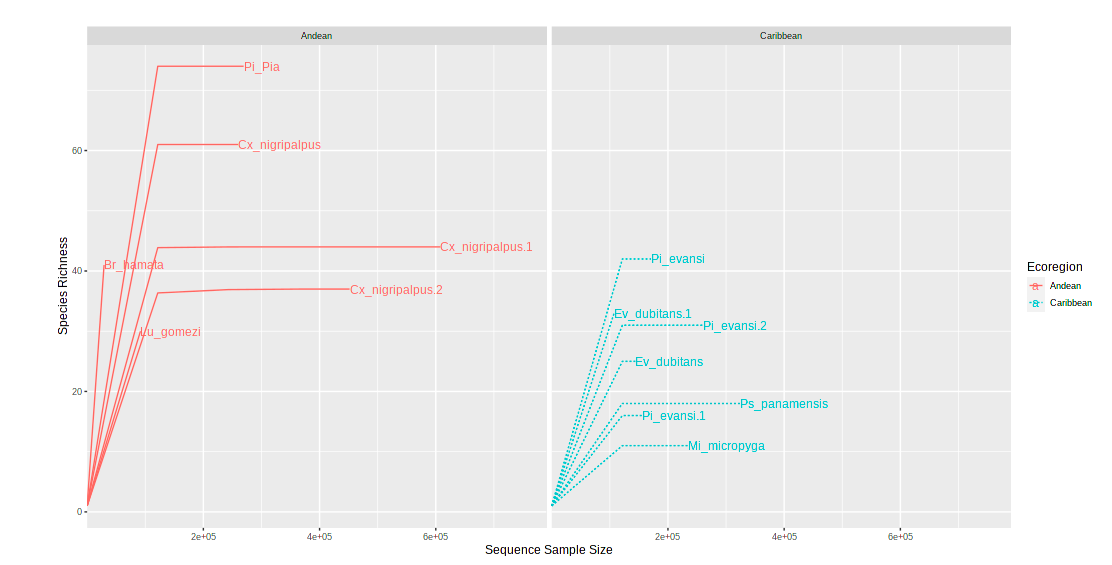

Supplement: S3 Fig — (DOCX) [file pntd.0009942.s005.docx]
