# Supplementary material for: Molecular phylogeny of heritable symbionts and microbiota diversity analysis in phlebotominae sand flies and Culex nigripalpus from Colombia
Source: PLoS Negl Trop Dis. 2021 Dec 20;15(12):e0009942. doi: 10.1371/journal.pntd.0009942 (PMC8722730; doi:10.1371/journal.pntd.0009942)
Supplement: S4 Fig — (DOCX) [file pntd.0009942.s006.docx]

**S4 Fig**. Gut microbiota composition in wild specimens of several natural population of phlebotominae sand flies and *Cx. nigripalpus*. Relative abundance of ASVs that were called to the taxonomic rank of Phylum.


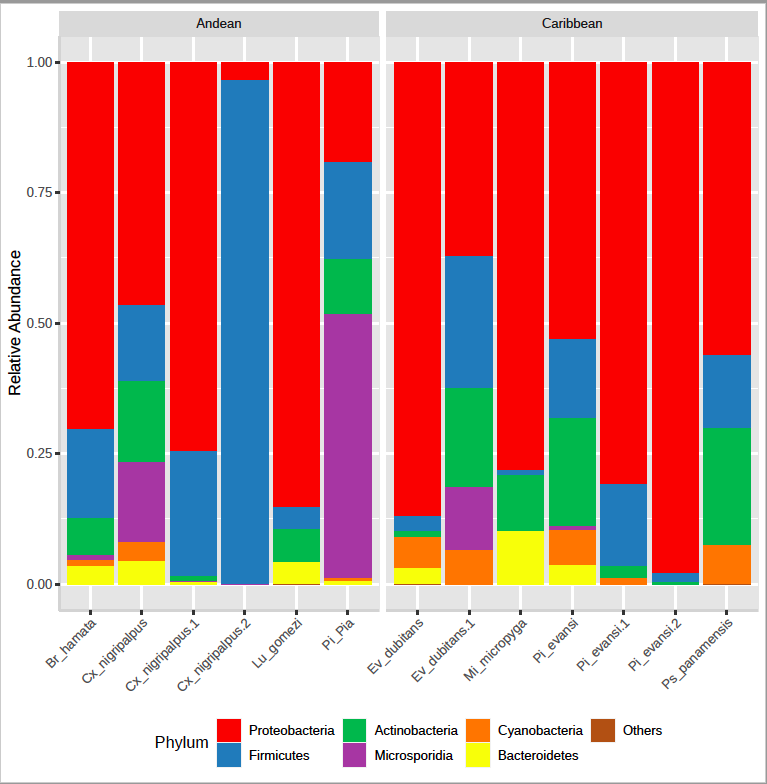


**a)**
