# Supplementary figures and images for: Molecular phylogeny of heritable symbionts and microbiota diversity analysis in phlebotominae sand flies and Culex nigripalpus from Colombia
Source: PLoS Negl Trop Dis. 2021 Dec 20;15(12):e0009942. doi: 10.1371/journal.pntd.0009942 (PMC8722730; doi:10.1371/journal.pntd.0009942)

**S5 Fig.** Core microbiotaof phlebotominae sand flies and *Cx. nigripalpus.*


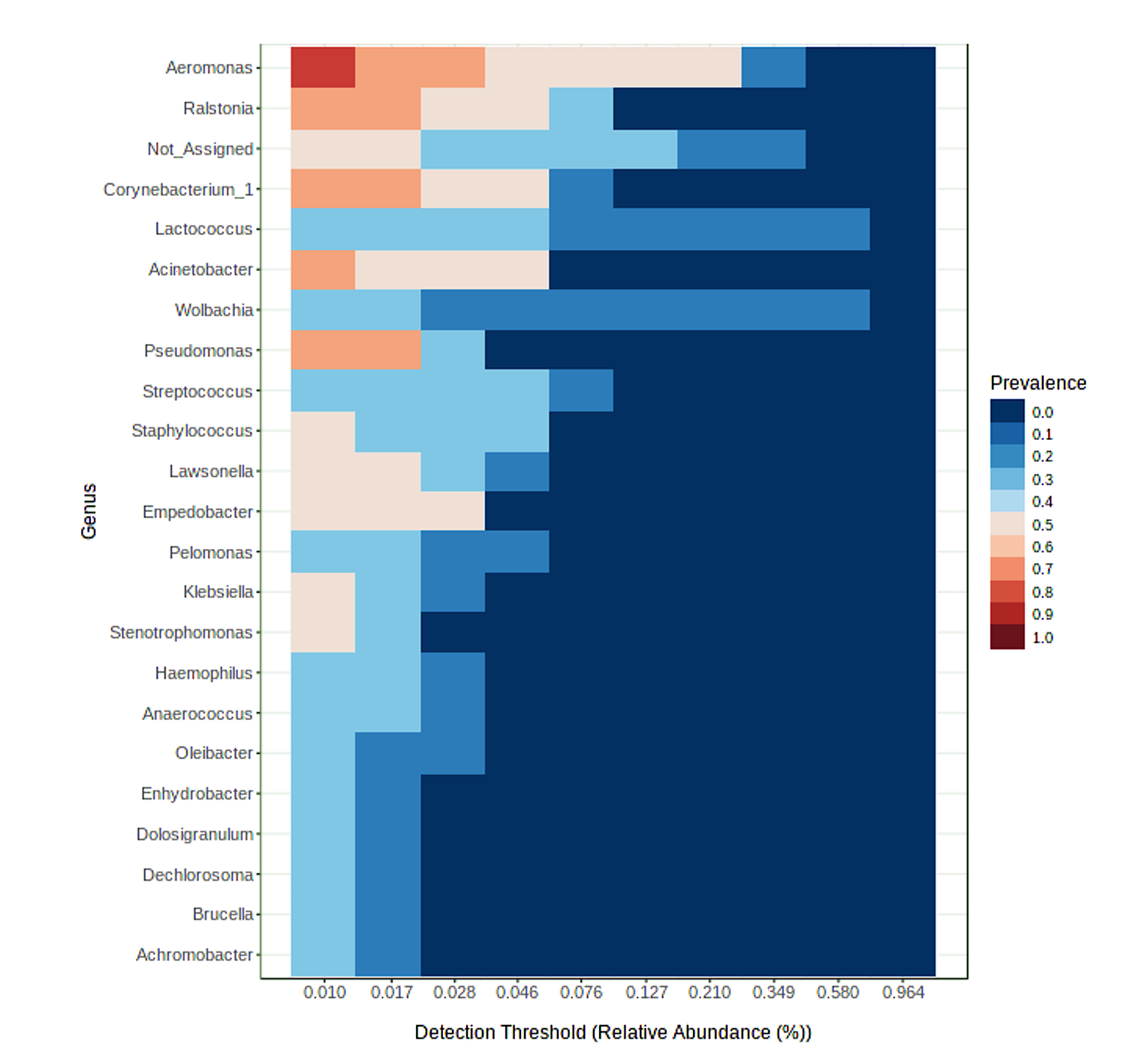

Supplement: S5 Fig — (DOCX) [file pntd.0009942.s007.docx]
